# From Solution to Surface: How the Catalytic Environment Modulates Peptide Bond Cleavage by Metal‐Oxo Cluster Nanozymes

**DOI:** 10.1002/advs.202519545

**Published:** 2026-01-05

**Authors:** Kilian Declerck, Muhammed Jibin Parammal, Carlotta Seno, Thomas J. N. Hooper, Dimitrios Sakellariou, Jonathan De Roo, Nada D. Savić, Tatjana N. Parac‐Vogt

**Affiliations:** ^1^ Department of Chemistry KU Leuven Leuven Belgium; ^2^ Institut Lavoisier De Versailles, CNRS, UVSQ Université Paris‐Saclay Versailles France; ^3^ Department of Chemistry University of Basel Basel Switzerland; ^4^ Centre for Membrane Separations, Adsorption, Catalysis and Spectroscopy for Sustainable Solutions (cMACS) KU Leuven Leuven Belgium

**Keywords:** heterogeneous, homogeneous, metal‐oxo clusters, protein hydrolysis

## Abstract

Nanozymes that selectively cleave proteins offer a promising alternative to natural proteases due to their superior stability, tunability, and scalability. However, they are either water‐soluble, preventing efficient recovery and limiting their practical application, or structurally ill‐defined and insoluble, hindering mechanistic understanding and rational catalyst design. To address this, we developed a structurally well‐defined dodecanuclear hafnium‐based metal‐oxo cluster with a tunable solubility that enables direct comparison of homogeneous and heterogeneous catalytic behavior in peptide bond hydrolysis. The soluble cluster, Hf_12_(sol), and its insoluble counterpart, Hf_12_(precip), share identical core structures according to pair distribution function analysis and possess highly similar ligand environments as indicated by solution‐ and solid‐state NMR as well as FT‐IR spectroscopy. We demonstrate that both forms efficiently cleave peptide bonds in the dipeptide glycylglycine and the more complex protein myoglobin. Solution‐based spectroscopic studies with Hf_12_(sol) show direct coordination of the peptide bond to Hf(IV) centers, with substrate stabilization via cooperative binding to the cluster, whereas Hf_12_(precip) shows reusability over multiple reaction cycles without loss of structural integrity. This highlights the potential of group IV metal‐oxo clusters as synthetic proteases and offers a rare platform to correlate molecular reactivity with macroscopic catalytic behavior across phases, thereby deepening our understanding of how proteolytic reactions can be modulated by catalyst structure and solubility.

## Graphical abstract

Comparison of homogeneous Hf_12_(sol) and heterogeneous Hf_12_(precip) metal‐oxo clusters enables investigation of how solubility influences nanozymatic reactivity and selectivity toward proteolysis, without introducing confounding factors from differences in cluster identity. While the water‐soluble cluster allows performing detailed mechanistic studies, the insoluble cluster can be recycled as catalyst for proteolysis and is easier to implement as nanozyme in proteomics applications.

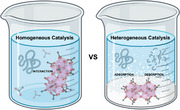

## 1 Introduction

Nanozymes, nanomaterials that mimic enzymatic activity, have become increasingly important in catalysis due to their stability, tunability, and scalability, offering key advantages over natural enzymes across a wide range of applications, from biosensing to disease treatment [1, 2, 3, 4, 5]. Most nanozymes, however, are structurally ill‐defined bulk materials, making it difficult to pinpoint active sites or understand reaction mechanisms, which limits their utility in mechanistic studies and rational catalyst design. To overcome these limitations, atomically precise systems have been developed that retain the nanozyme‐like activity while providing well‐defined structures [6]. In this context, catalysts based on hard Lewis acid metal ions are particularly effective due to their strong affinity for oxygen‐donor ligands, like those in carbonyl or phosphate groups, which enhances the electrophilicity of adjacent atoms [7, 8]. As a result, otherwise stable bonds become susceptible to transformations such as hydrolysis or substitution [9, 10]. One important example is the peptide bond in proteins, an amide linkage whose resonance stabilization renders it extremely resistant to hydrolysis under physiological conditions [11, 12]. Making nanozymes that are able to act as synthetic proteases is particularly important for the field of proteomics, where it would enable more efficient analysis of the protein structure, modifications, and interactions through targeted peptide generation [13, 14, 15, 16].

Coordination of a Lewis acid to the carbonyl oxygen disrupts the resonance of the amide bond, thereby increasing the susceptibility of the carbonyl carbon to a nucleophilic attack of water, a key step in metal‐catalyzed proteolysis [13, 16]. Group IV metals such as zirconium and hafnium, with a high coordination number and strong oxophilicity, can form well‐defined, polynuclear metal‐oxo clusters (MOCs) via olation and oxolation reactions that are stabilized and extracted in the presence of coordinating anionic ligands like carboxylates and phosphinates [17, 18]. These polynuclear MOCs have been proven to catalyze hydrolysis reactions as a result of their Lewis acidity and are more thermodynamically and kinetically stable compared to metal salts due to their rigid, interconnected structure and ligand shell [19, 20, 21, 22, 23, 24]. In addition, MOCs feature multiple closely spaced Lewis acid sites that enable cooperative binding of multidentate substrates such as proteins, which can induce a bias toward particular peptide sequences depending on the cluster core geometry and nuclearity [20, 25]. As a result, MOCs have emerged as promising class of catalysts for selective peptide bond hydrolysis, offering advantages in tunability and robustness over enzymatic or small‐molecule approaches [14].

The capping ligands surrounding the clusters can be exchanged easily, allowing their surface properties to be tuned [18, 26, 27, 28]. This provides the means to influence the substrate‐cluster interactions, for example by modifying the charge and polarity of the clusters [14]. It also enables the generation of MOCs with open metal sites by controlled removal of one or more capping ligands, thereby increasing active site accessibility and enhancing reactivity [20, 23, 29]. However, most importantly, the solubility of MOCs can be adapted by carefully selecting capping ligands and/or counterions, providing us with an excellent opportunity to study them as both homogeneous and heterogeneous catalysts [17]. Soluble MOCs can be studied using solution‐based analytical techniques, allowing precise characterization of their structure, speciation, and interactions with proteins [20]. However, their solubility in water generally prevents efficient recovery or reuse, limiting their practical application. Insoluble MOCs, on the other hand, offer the advantage of easy physical separation and potential recyclability [19, 30, 31, 32]. However, although insoluble catalysts offer improved durability during repeated use and are well‐suited for applications requiring clean separation from the product, their solid‐state nature makes mechanistic characterization of the catalyst and its interactions with the proteins significantly more challenging. The tunable solubility of MOCs thus enables direct comparison of their reactivity and selectivity in solution and at solid‐liquid interfaces, providing insight into whether the peptide bond hydrolysis follows similar mechanisms in both situations. These types of correlations are uncommon in catalysis and may provide important perspective on how to better control proteolysis.

To enable the direct comparison of soluble and insoluble metal‐based catalysts with identical active sites, we designed a Hf‐based metal‐oxo cluster with tunable solubility where the cluster core structure is preserved, and the ligand environment is maintained with minimal variation. Specifically, we synthesized the Hf_6_O_4_(OH)_4_(CH_3_CO_2_)_7_Cl_4_^+^ (Hf_6_) cluster, a hexanuclear cluster previously developed using a Zr(IV)‐based analogue [33]. Similarly, the Hf_6_ cluster dimerizes after incubation at elevated temperature in aqueous solutions, which results in the formation of an oxygen‐bridged Hf_12_ species, hereafter referred to as Hf_12_(sol) [20]. Notably, by adjusting the pH of the solution using a base, we were able to isolate an insoluble form of this dimer, referred to as Hf_12_(precip). This material is structurally identical to the soluble version of the Hf_12_ cluster with respect to metal‐oxo connectivity and associated ligands, providing a unique system to directly compare the solution‐phase and solid‐phase reactivity of the same cluster catalyst. This model system was applied to study the hydrolysis of peptide bonds in both glycylglycine (GG), a simple model dipeptide, as well as myoglobin (Mb), a complex globular protein. These substrates allowed us to probe the catalytic activity across different levels of structural complexity, where the interactions with GG gave detailed insights into molecular‐level interactions leading to the hydrolysis of the peptide bond, while Mb hydrolysis allowed comparison of cleavage selectivity. Hf_12_(sol) could be easily characterized using solution‐based techniques, whereas Hf_12_(precip) retained its catalytic activity and could be easily recovered and reused after the reaction. This allowed us to correlate molecular‐level insights obtained from the homogeneous system with the practical utility of a recyclable, heterogeneous analogue, shedding light on how structural features of the catalyst translate into functional performance across different physical states.

## 2 Structural Characterization and Speciation of Soluble and Insoluble Hf‐Oxo Clusters

### 2.1 Synthesis and Characterization of a Hf_6_ Cluster

**FIGURE 1:**
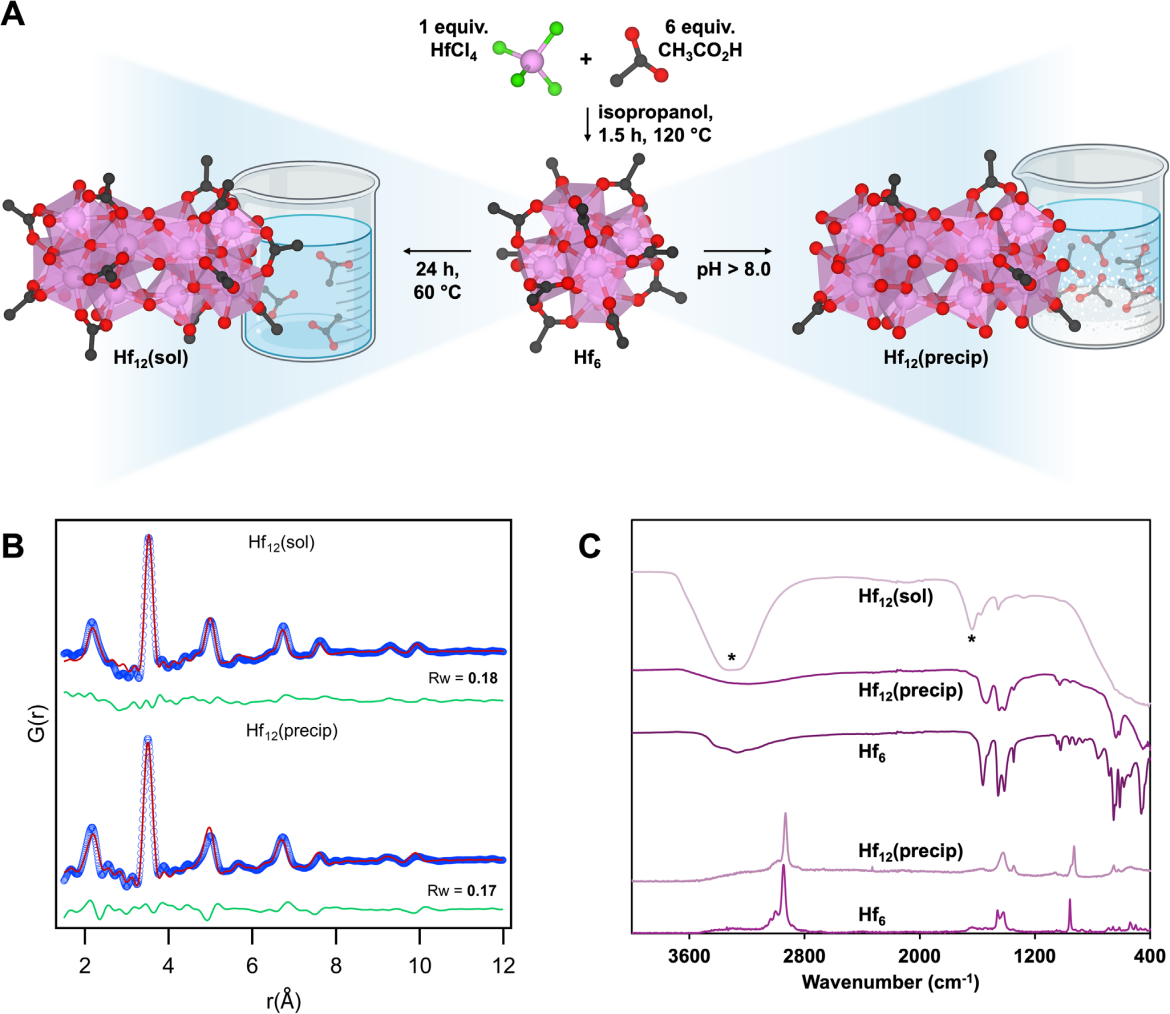
(A) Synthesis of the Hf_6_ precursor by mixing a 1:6 molar ratio of HfCl_4_ and acetic acid in isopropanol. Hf_12_(sol) is synthesized by incubating Hf_6_ in water at 60°C for 24 h. Hf_12_(precip) is obtained by adding 1.0 M NaOH to an aqueous Hf_6_ solution until pH 8.0 is reached. (B) PDF fits of Hf_12_(sol) and Hf_12_(precip) with an oxygen‐bridged Hf_12_ model structure extracted from CCDC 2002902 [36]. Refining parameters are given in Table S3. (C) Normalized FT‐IR (transmittance) and Raman (intensity) spectra of Hf_12_(precip) and Hf_6_, indicating a reduction in acetate ligands. The FT‐IR spectrum of 100 mM Hf_12_(sol) in water (*) retains the peaks corresponding to acetate.

A new hexanuclear hafnium‐based cluster, an isostructural analogue of the previously reported Zr_6_O_4_(OH)_4_(CH_3_CO_2_)_8_(H_2_O)_2_Cl_3_^+^ (Zr_6_) cluster, was synthesized using a modified procedure in which HfCl_4_ was used instead of ZrCl_4_ [20, 33]. To ensure an identical cluster nuclearity and a comparable coordination environment, HfCl_4_ was combined with acetic acid in isopropanol at a 1:6 molar ratio, identical to the synthesis of Zr_6_ (Figure 1A) [33]. As literature data indicate that Hf‐oxo clusters are more challenging to synthesize than their Zr‐oxo analogues, requiring higher temperatures and longer reaction times, the mixture was incubated for 1.5 h rather than 1 h to ensure comparable yields to the Zr_6_ synthesis [24, 25]. The product was characterized by powder X‐ray diffraction (PXRD), Fourier transform infrared (FT‐IR) spectroscopy, and high‐resolution mass spectrometry (HR‐MS). PXRD confirmed that the Hf_6_ cluster is crystalline, displaying a diffraction pattern closely matching that of the previously reported Zr(IV) analogue, suggesting they share the same long‐range crystalline structure and phase composition (Figure S1A) [20]. Moreover, pair distribution function (PDF) analysis validated the presence of a similar hexanuclear core structure (Figure S2). FT‐IR spectroscopy further confirmed the presence of acetate capping ligands with characteristic peaks corresponding to asymmetric COO^−^ (ν_as_ ∼ 1550 cm^−1^) and symmetric COO^−^ (ν_s_ ∼ 1430 cm^−1^) stretching vibrations. A band separation of less than 200 cm^−1^ indicates bidentate coordination of the acetate ligands (Figure S1B) [34]. HR‐MS analysis was performed immediately after dissolution of the Hf_6_ cluster in water and revealed a species consistent with the molecular formula Hf_6_O_4_(OH)_4_(CH_3_CO_2_)_7_Cl_4_^+^ (Figure S3). Therefore, the primary structural difference between the Zr_6_ and Hf_6_ clusters lies in the number and, to a lesser extent, the nature of the capping ligands coordinated to an otherwise identical cluster core [20, 33]. This suggests that charge neutrality of the solid Hf_6_ cluster is maintained by the presence of an additional coordinated chloride or hydroxide ion [35].

### 2.2 Structural Integrity and Transformation of the Hf_6_ Cluster

The stability and structural integrity of the Hf_6_ cluster were evaluated under a range of reaction conditions, including elevated temperatures and pH, designed to mimic environments typically encountered during hydrolysis reactions, where catalytic activity is optimal. Incubation of Hf_6_ in aqueous solution at 60°C for 24 h resulted in behavior comparable to that of the isostructural Zr_6_ cluster under the same conditions where (1) partial exchange of capping ligands with the solvent leads to displacement of acetate ligands, and (2) an oxygen‐bridged dimer forms as a result of this ligand displacement [20]. Ligand exchange was confirmed by ^1^H nuclear magnetic resonance (NMR) spectroscopy, which showed that 53.92% of bound acetate was released after 24 h (Figure S4A,C). In addition, ^13^C NMR revealed three characteristic peaks that can be assigned to the Hf(IV)‐coordinated acetate ligands at 26.6, 182.3, and 182.9 ppm, along with two additional peaks appearing after incubation at 23.5 and 180.0 ppm indicative of free acetic acid (Figure S4B). This perturbation in the cluster's coordination environment further led to the formation of an oxygen‐bridged dimer, as proven by PDF analysis. The model of an oxygen‐bridged dodecanuclear cluster (extracted from CCDC 2002902 [36]) provided a good fit (R_W_ = 0.18) for an aqueous solution containing 10 mm Hf_6_ after 24 h at 60°C (Figure 1B). This was further substantiated by diffusion ordered spectroscopy, which confirmed that the monomeric Hf_6_ cluster dimerized after 24 h at 60°C (Figure S5).

Raising the pH of an aqueous solution containing either the Hf_6_ cluster (0 h) or Hf_12_(sol) (24 h, 60°C) by addition of 1.0 M NaOH led to precipitation in both cases when the pH surpassed 8.0. To evaluate the structural integrity of the resulting precipitate across the pH range 8.0–12.0 and to characterize its relation to the original cluster, a range of complementary techniques were employed, including PDF, FT‐IR, and Raman spectroscopy, transmission electron microscopy paired with energy dispersive X‐ray spectroscopy (TEM‐EDS), and thermogravimetric analysis (TGA). Structural modeling of the PDF data of the precipitate was performed using cluster models of varying nuclearity, ranging from 6 to 18. The best fit (R_W_ = 0.17) was obtained using the dodecanuclear oxygen‐bridged Hf_12_ model, indicating that the precipitate contains Hf_12_ units with the same local structure as observed after incubation of the Hf_6_ precursor at 60°C (Figure 1B). We therefore refer to the soluble dimer as Hf_12_(sol) and the precipitated form as Hf_12_(precip). While the Hf_12_ units in Hf_12_(precip) remain coherent, PDF cannot resolve whether they are associated in extended or random assemblies. Although minor impurities of amorphous Hf(OH)_4_ cannot be fully excluded, the absence of features in the PDF typical for gel‐like metal hydroxides/oxides indicates that HfO_2_‐like and substantial Hf(OH)_4_‐like species are not present. This conclusion is further supported by Raman spectroscopy, which corroborated the lack of such phases (Figure S6) [37, 38, 39].

The morphology of the precipitate was investigated using TEM, revealing individual particles with an average diameter of 4.93 ± 0.52 nm (Figure S7). The observed particles are significantly larger than the theoretical size of an isolated Hf_12_ cluster, which is approximately 1.5 nm (based on the model xyz file provided in the Supplementary Information), indicating that these particles are formed by aggregation. However, no other large‐scale supramolecular assemblies beyond this contained aggregate were observed by TEM, which suggests that the insolubility of Hf_12_(precip) is inherent to the material and may also be caused by subtle changes in surface chemistry associated with the coordination environment. To characterize the ligand shell surrounding Hf_12_(precip), FT‐IR and Raman spectroscopy, along with EDS were used. However, both the FT‐IR and Raman spectra of Hf_12_(precip) closely resembled those of Hf_6_ and Hf_12_(sol) (Figure 1C), displaying the same characteristic peaks that are associated with coordinated acetate ligands. The intensity of peaks corresponding to acetate was significantly reduced for Hf_12_(precip), indicating partial release during precipitation. To quantify the extent of the ligand loss, NMR spectroscopy was used. 1.0 m NaOD was added to 5.0 mm Hf_6_ in D_2_O to induce precipitation. ^1^H NMR analysis of the resulting supernatant showed that, depending on the pH (8.0–12.0), 38.57%–48.64% of acetate ligands remained in solution (Figure S8). Hf_12_(sol) showed an acetate loss of 53.92% after 24 h at 60°C, which is closely comparable to the maximum ligand loss observed for Hf_12_(precip). EDS further confirmed the presence of chloride ligands in Hf_12_(precip) and also revealed the presence of sodium, attributed to NaOH from the precipitation process (Figure S9). Therefore, chloride can be either directly coordinated to Hf(IV) centers or present as the byproduct NaCl. We used the Spectroquant Chloride Test, a commercially available photometric assay, for quantification of chloride ligand loss, which revealed that Hf_12_(precip) contains 4.43 wt.% chloride while Hf_12_(sol) contains 6.73 wt.% chloride (Figure S10). Hf_12_(precip) contains approx. 34% less chloride in comparison to Hf_12_(sol), thus further supporting the earlier conclusion that the ligand environment of the Hf(IV) centers was altered by precipitation.

To quantify the ligand content further, Hf_12_(precip) was characterized by TGA. This revealed a residual mass of 68.31% at 600°C for Hf_12_(precip), which is slightly higher than the residual mass of 65.78% for the Hf_6_ cluster (Figure S11). This indicates that Hf_12_(precip) possesses a higher proportion of inorganic content corresponding to the cluster core, consistent with a reduced number of acetate and chloride ligands. Finally, to assess whether the Hf(IV) content decreased after precipitation of Hf_12_(precip), and thus whether the precipitation was complete, the supernatant obtained after precipitation at pH 8.0–12.0 was analyzed using inductively coupled plasma optical emission spectroscopy (ICP‐OES) (Table S1). Negligible amounts of Hf(IV) were detected (< 0.1%) under all evaluated conditions, indicating that conversion of Hf_6_ to Hf_12_(precip) was complete at pH > 8.0. Since the concentration of acetate and chloride ligands is reduced and the Hf(IV) content remains unchanged, it is most likely that the core of Hf_12_(precip) is capped by residual acetate and chloride ligands as well as water and hydroxide ligands to ensure charge neutrality and a saturated coordination environment. We can determine the ligand shell population surrounding Hf_12_(precip) quantitatively by calculating the minimal formula according to Pulparayil Mathew et al. [40]. (calculations provided in the Supplementary Information). The experimental molar mass of Hf_12_(precip) (3697.67 g/mol) could be calculated from TGA (Figure S11), and the number of acetate and chloride ligands could be determined from ^1^H NMR spectroscopy (Figure S12) and a photometric chloride test (Figure S10), respectively, after digesting Hf_12_(precip) with 1.0 M NH_4_HCO_3_. After confirming charge balance and coordination saturation of the Hf(IV) centers, Hf_12_(µ_3_‐O)_8_(µ_3_‐OH)_8_(µ_2_‐OH)_6_(CH_3_COO)_5.5_(Cl)_4.7_(OH)_7.8_(H_2_O)_12.5_ was assigned as the minimal formula of Hf_12_(precip).

## 3 Protease Activity of Soluble vs. Insoluble Hf_12_ Clusters

### 3.1 Mechanistic Perspectives From Dipeptide Hydrolysis

**FIGURE 2:**
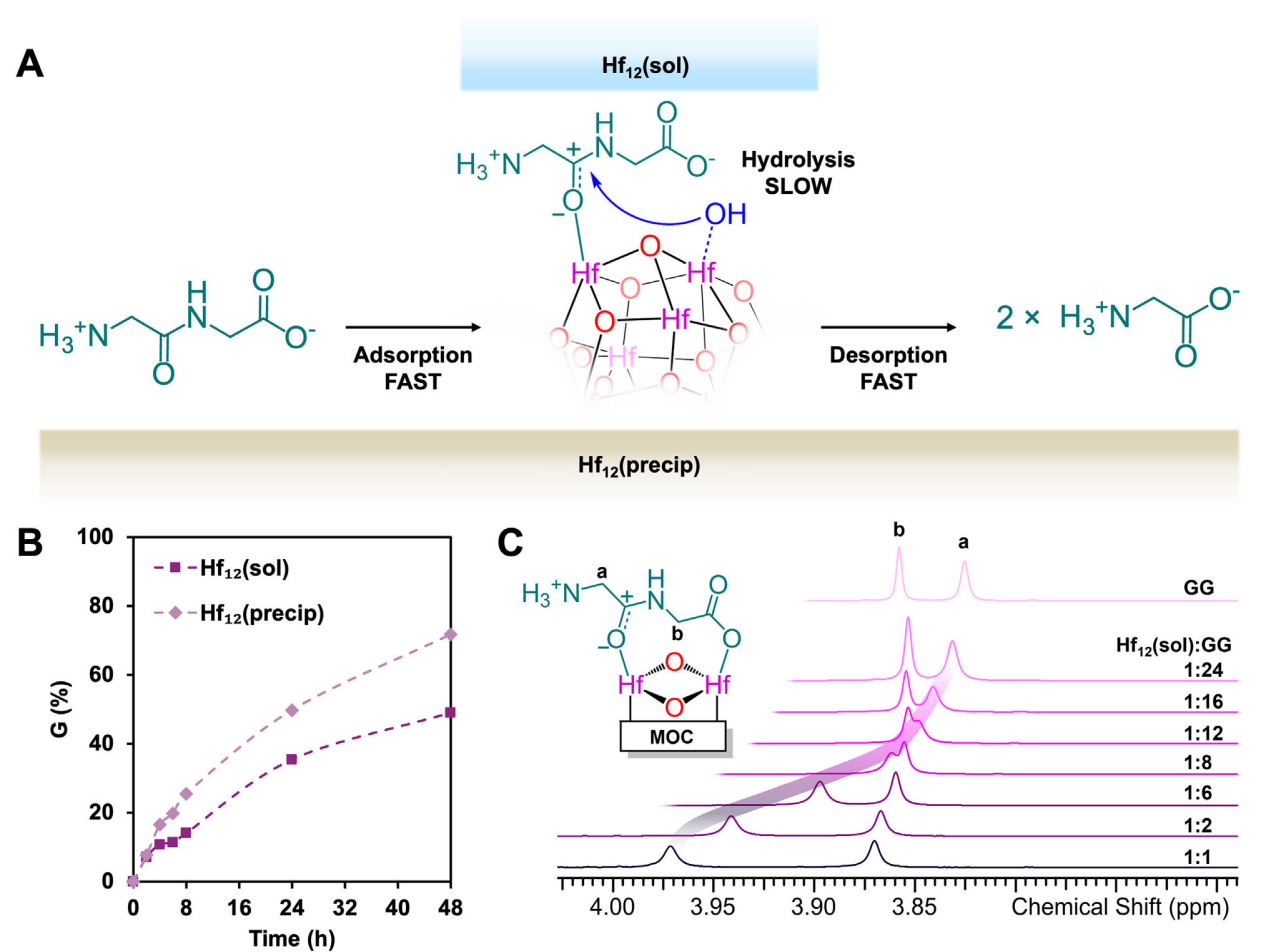
(A) Schematic representation of the interaction between glycylglycine (GG) and both forms of the Hf_12_ cluster. (B) Comparison of glycine (G) production with both clusters over time. (C) ^1^H NMR spectra of 2.0 mM Hf_12_(sol) and 2.0–48.0 mM GG exhibiting deshielding of both CH_2_ peaks, with more pronounced deshielding observed for the N‐terminal CH_2_ group, suggesting coordination through the peptide bond and carboxyl group oxygen atoms. ^1^H NMR spectrum of 2.0 mM GG is shown on top.

The reactivity of both clusters toward peptide bond activation and subsequent hydrolysis in aqueous solution was assessed as a useful benchmark to evaluate the effect of variations in the ligand environment and cluster solubility on the catalytic efficiency (Figure 2A). Solutions containing 2.0 mm Hf_12_(sol), adjusted to pD 7.4 using 1.0 m NaOD, or 2.0 µmol Hf_12_(precip), which naturally exhibits a pD of 7.4, and 2.0 mm GG were incubated in 1.0 mL D_2_O at 60°C. Peptide bond hydrolysis was monitored by ^1^H NMR spectroscopy (Figure S13A,B). Hydrolysis of GG into glycine (G) followed first‐order kinetics for both catalysts, resulting in rate constants of 5.44 × 10^−6^ s^−1^ (t_½_ = 35.4 h) for Hf_12_(sol) and 6.56 × 10^−6^ s^−1^ (t_½_ = 29.4 h) for Hf_12_(precip) based on the disappearance of GG (Figure 2B; Figure S13C,D). This corresponds to conversions of 49.1 and 71.7% after 48 h, representing a substantial rate enhancement relative to the uncatalyzed reaction (6.3 × 10^−11^ s^−1^, t_½_ = 350 years) [11]. In addition, these rates closely resemble those observed for the previously studied Zr_6_ cluster (t_½_ = 23.0 h). To assess how the coordination environment and metal ion identity affect substrate‐cluster interactions, the binding mode of GG to Hf_12_(sol) was characterized using an approach similar to previously reported ones [20]. Specifically, 2.0 mM Hf_12_(sol) was incubated with 2.0–48.0 mM (1–24 equiv.) GG at 60 °C for 2 h without pH adjustment (pD 5.0, selected to minimize hydrolysis and compare to a similar experiment with Zr_6_), followed by analysis via ^1^H NMR spectroscopy. The results indicate that Hf_12_(sol) coordinates GG through the peptide bond oxygen, facilitating activation toward hydrolysis, while additionally stabilizing the intermediate by coordination to the carboxyl‐group oxygen, which is deprotonated at pD 5.0 (Figure 2C). An identical coordination geometry between the Zr_6_ dimer and GG was observed, which likely accounts for the similarities in their hydrolytic performance.

Interestingly, Hf_12_(precip) hydrolyzes GG more efficiently than Hf_12_(sol). This difference in the reactivity could be attributed to the partial loss of acetate ligands (48.64%) during precipitation of Hf_12_(precip). While the acetate ligands released by Hf_12_(precip) are removed with the supernatant before hydrolysis reactions, acetate ligands released upon formation of Hf_12_(sol) remain in the reaction medium. Therefore, although ligand exchange with the solvent occurs for both clusters, as confirmed by ^1^H NMR (Figures S4 and S14), a higher concentration of acetic acid in the presence of Hf_12_(sol) may hinder substrate binding through competitive inhibition. This ultimately reduces the reactivity of the soluble cluster relative to its insoluble counterpart. Additionally, a lower concentration of acetic acid facilitates the coordination of hydroxide ions to the cluster, positioning them in close proximity to the active site where they can readily participate in nucleophilic attack on coordinated peptide bonds and facilitate their cleavage. These results highlight the critical role of controlled ligand modulation around the cluster core in balancing ligand exchange, which aids in improving the catalytic activity without compromising structural stability.

To gain deeper insight into the differences in reactivity of the two Hf_12_ clusters toward peptide bond hydrolysis, the time‐dependent adsorption of GG on Hf_12_(precip) was monitored. This analysis provides valuable information on the substrate's interaction with the catalyst surface. Therefore, we investigated the time‐dependent adsorption capacity of Hf_12_(precip) by incubating 2.0 mm GG and 2.0 µmol insoluble cluster in 1.0 mL D_2_O at 25°C to prevent hydrolysis (Figure S15). While GG adsorption gradually increased over time, only 8.91% was retained on the surface of Hf_12_(precip) after 8 h of incubation. This may indicate rapid and reversible substrate binding, where GG adsorption is balanced by an equally fast desorption process. The dynamic nature of the exchange process causes an increased number of individual binding events of GG to Hf(IV) centers, thereby enhancing the overall reaction rate. This further indicates that both adsorption of GG and desorption of G do not limit the rate of the reaction. Instead, the hydrolysis reaction itself is most likely the rate‐limiting step, which is corroborated by the faster consumption of GG relative to the formation of G (Figure S16). Therefore, it is more accurate to describe the observed phenomenon as substrate retention rather than true adsorption capacity in this context, since transient binding of GG is essential for hydrolysis to occur.

To confirm that the high reactivity of Hf_12_(precip) arises from a well‐defined molecular structure, rather than solely from the presence of the Hf(IV) ions, control experiments were performed using alternative Hf(IV)‐containing precipitates. These samples were prepared by dissolving Hf salts (HfCl_4_ or HfOCl_2_) followed by precipitation under the same conditions used for preparation of Hf_12_(precip) (pH > 8.0). The resulting precipitates were incubated with 2.0 mm GG at 60°C, maintaining a Hf(IV) concentration of 24.0 mm, which is equivalent to 2.0 µmol Hf_12_(precip) in 1.0 mL to allow for direct comparison of the catalytic activity (Figure S17). Under these conditions, only 7.68% and 7.44% of GG was hydrolyzed to G by the HfCl_4_‐ and HfOCl_2_‐derived precipitates, respectively, compared to 49.79% using Hf_12_(precip) after 24 h. Raman spectroscopy (Figure S6) and PDF analysis (Figure S18) confirmed that the precipitates obtained from HfCl_4_ and HfOCl_2_ differ substantially from Hf_12_(precip). These results thus highlight the critical role of the specific molecular structure of Hf_12_(precip) beyond the mere presence of Hf(IV) ions.

### 3.2 Recyclability of Insoluble Hf_12_ Cluster for Dipeptide Hydrolysis

**FIGURE 3:**
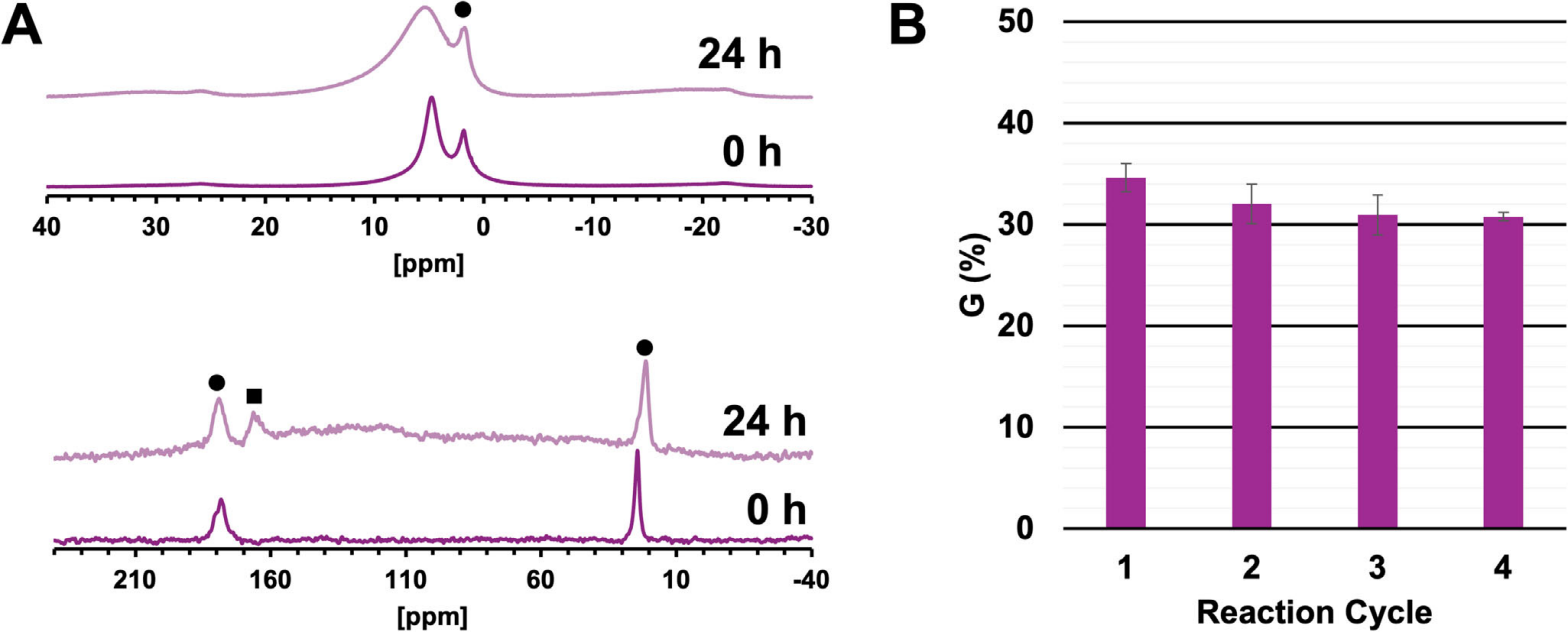
(A) Solid‐state ^1^H (top) and ^13^C (bottom) NMR spectra of Hf_12_(precip) directly after synthesis and after incubation in water at 60°C for 24 h. Hf(IV)‐coordinated acetate is denoted by ● and ▪ indicates non‐coordinated acetic acid. (B) Conversion of 2.0 mM GG to G after 12 h at 60°C in the presence of 2.0 µmol Hf_12_(precip) in 1.0 mL D_2_O for four reaction cycles. Water (24 h, 60°C) was used to remove excess GG/G between cycles. Measurements were performed in triplicate. Error bars represent the standard deviation.

A combination of high reactivity and low substrate/product retention suggests that Hf_12_(precip) is less prone to catalyst poisoning and thus well‐suited for recycling. Consequently, the structural and chemical stability of Hf_12_(precip) is particularly important. The stability of the ligand shell was characterized by solid‐state NMR spectroscopy, whereas ICP‐OES confirmed the phase stability of the inorganic Hf(IV)‐oxo core. The ^1^H ssNMR spectra revealed a decrease in acetic acid content within Hf_12_(precip) from 9.73 to 2.10 wt.% after 24 h in water at 60°C (Figure 3A). The acetic acid content decreases further to 1.71 wt.% upon incubation with GG, consistent with substrate‐induced ligand exchange. In addition, ^13^C ssNMR of Hf_12_(precip) showed two resonances at 24.4 and 178.3 ppm, which can be attributed to Hf(IV)‐coordinated acetate ligands. Following incubation, a new signal emerged at 166.4 ppm (Figure 3A), most likely corresponding to non‐coordinated (free) acetic acid associated with the solid phase through hydrogen bonding or physisorption onto the surface of the material. Next, ICP‐OES was used to quantify the Hf(IV) concentration in the supernatant after incubating Hf_12_(precip) between pH 2.0 and 12.0 to assess potential leaching. Following 144 h at 60°C, the maximum detected Hf(IV) concentration corresponded to less than 0.1% of the initial Hf_12_(precip) mass, indicating negligible re‐dissolution and confirming the material's stability under the investigated reaction conditions (Table S2).

Due to the high structural stability of Hf_12_(precip), its reusability as an insoluble catalyst in peptide bond hydrolysis was evaluated over multiple cycles of GG hydrolysis. Unlike metal‐organic frameworks (MOFs), clusters are non‐porous materials and do not require activation prior to catalysis. As a result, regeneration of the cluster by simple water washing between cycles does not compromise its structural integrity. Moreover, an absence of solvent‐accessible pores in Hf_12_(precip) eliminates the risk of structural collapse during drying commonly induced by water's high surface tension, which would otherwise block catalytically active sites [30, 31]. Nonetheless, a slight decline in catalytic activity was observed, with the yield of G decreasing from 34.6% in the first cycle to 31.8% in the fourth cycle after 12 h of reaction time (Figure 3B). This slight reduction in reactivity is likely a result of surface passivation or non‐covalent aggregation of individual cluster units. Although Hf_12_(precip) shows a modest drop in activity upon recycling, its overall reactivity exceeds by far that of Hf‐NU‐1000 (>30% GG hydrolyzed after 12 h vs. 20%–23% after 24 h) even though the latter retains full activity over five cycles. These results demonstrate that Hf_12_(precip) maintains high catalytic activity over multiple cycles, highlighting its potential as a robust and recyclable catalyst for peptide bond hydrolysis under a wide range of conditions.

### 3.3 Protein Hydrolysis: Reactivity and Selectivity of Hf_12_ Clusters Toward Myoglobin

**FIGURE 4:**
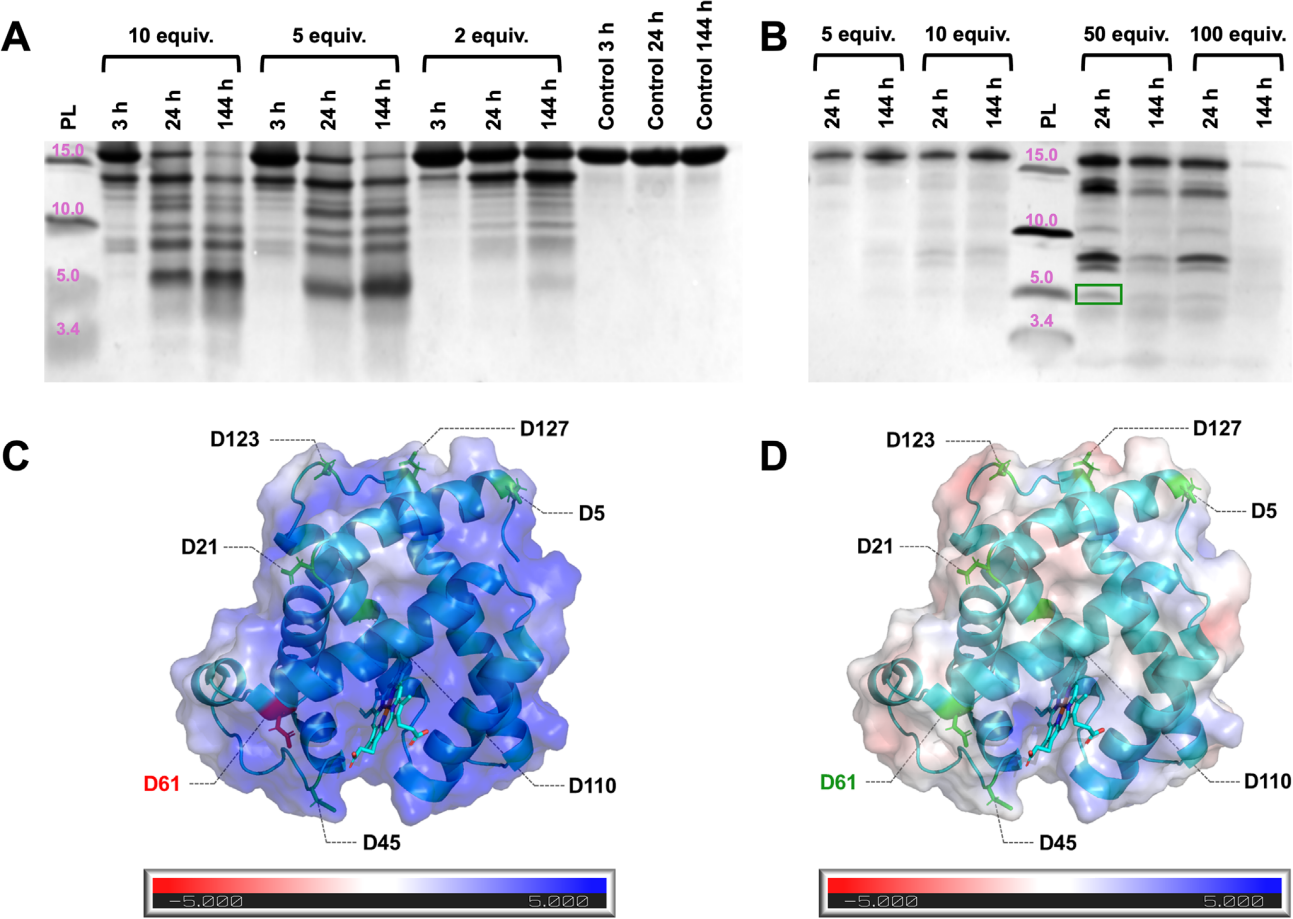
Silver‐stained SDS‐PAGE results of hydrolysis of 0.02 mm Mb at 60°C by (A) 0.04–0.2 mm (2–10 equiv.) Hf_12_(sol) and (B) 0.1–2.0 µmol (5–100 equiv.) Hf_12_(precip) in 1.0 mL of water. Fragment generated by cleavage at D61 is highlighted in green. Surface charge model of Mb (PDB 1WLA) in the presence of (C) Hf_12_(sol) at pH 4.5 and (D) Hf_12_(precip) at pH 7.4. Aspartate (D) residues are highlighted in green. D61, near the heme group, resides within the most positively charged region at pH 4.5, highlighted in red.

The reactivity of Hf_12_(sol) and Hf_12_(precip) toward peptide bond cleavage was further evaluated using a more complex substrate: equine skeletal muscle myoglobin (Mb), a 153‐amino acid protein. Mb hydrolysis was previously investigated in the presence of the soluble, isostructural Zr_6_ dimer, formed during incubation of the Zr_6_ cluster at elevated temperature [20]. Therefore, to evaluate the influence of the metal center as well as cluster solubility on proteolysis, 0.02 mm Mb was incubated at 60°C for up to 144 h with 0.04–0.2 mm (2–10 equiv.) Hf_12_(sol) or 0.1–2.0 µmol (5–100 equiv.) Hf_12_(precip) in 1.0 mL, where hydrolysis was followed by sodium dodecyl sulfate polyacrylamide gel electrophoresis (SDS‐PAGE). This revealed that Hf_12_(sol) cleaved Mb with a hydrolysis efficiency of 56.4–87.2% after 24 h, depending on the applied catalyst concentration (Figure 4A). The hydrolysis efficiency is comparable to that of the Zr_6_ dimer, which reached a maximum of approx. 95% after 24 h [20]. The high efficiency can be attributed to partial unfolding of Mb induced by Hf_12_(sol), similar to the Zr_6_ dimer, as evidenced by UV–vis, fluorescence, and circular dichroism spectroscopy (Figures S19–S21). These alterations in the protein structure facilitate interaction, thereby improving the hydrolysis efficiency.

Hydrolysis reactions in the presence of Hf_12_(precip), on the other hand, required higher catalyst loadings to generate the cleavage fragments at detectable concentrations. This observation is consistent with expectations, as Hf_12_(precip) exists as a larger precipitate in solution rather than a suspension, thus restricting its accessibility and interaction with the protein relative to Hf_12_(sol). Analysis by SDS‐PAGE showed that Hf_12_(precip) hydrolyzed Mb with an efficiency of 3.6%–64.4% after 24 h, depending on the catalyst concentration (Figure 4B). Notably, the highest hydrolysis efficiency was observed at 50 equiv. Hf_12_(precip). Increasing the catalyst loading to 100 equiv. resulted in a significant reduction in the intensity of both the peptide fragments and intact Mb over time. This non‐linear trend is likely attributed to protein aggregation induced by an excess of clusters, preventing required protein‐cluster interactions for hydrolysis to occur. Additionally, saturation of the cluster's surface caused by excessive protein adsorption would lead to similar results. Therefore, the adsorption of Mb onto the surface of Hf_12_(precip) was investigated by Bradford assay of the supernatant following incubation of 1.0–2.0 µmol Hf_12_(precip) with 0.02 mM Mb in 1.0 mL of water at 25°C for up to 12 h (Figure S22). Mb adsorption onto Hf_12_(precip) was found to range from 1.75% to 12.26%, which corresponds to 1 protein adsorbed for every 816–2857 units of Hf_12_(precip). This minimal protein adsorption likely contributes to the reduced hydrolysis efficiency. These results support the first hypothesis, i.e., extensive protein aggregation hinders effective interactions between the protein and cluster. An overview of the hydrolysis efficiency and relative abundance of individual fragments generated by both catalysts is provided in Table S4. Differences in catalyst loading limit direct comparison of the hydrolysis efficiency, so SDS‐PAGE can provide more meaningful insight through assessment of the cleavage selectivity.

Hydrolysis of Mb by Hf_12_(sol) resulted in the formation of 6 fragments with molecular weights of approx. 5.1, 7.6, 9.0, 10.4, 11.7, and 13.4 kDa (Figure 4A). Considering that the Zr_6_ dimer produced highly similar fragments, these results suggest similar interactions with the protein [20]. On the other hand, Hf_12_(precip) generated 6 fragments, but with molecular weights of approx. 5.1, 6.4, 7.6, 10.2, 11.7, and 13.4 kDa, closely matching most fragments produced by Hf_12_(sol), indicating that both catalysts cleave at similar sites within the protein (Figure 4B). Table S5 illustrates the similarities between the cleavage patterns obtained for the Zr_6_ dimer and both variants of the Hf_12_ cluster, supporting the conclusion that Hf_12_(sol) and Hf_12_(precip) hydrolyze the protein with a selectivity toward aspartate residues in the protein sequence. Although the majority of peptide fragments produced by Hf_12_(sol) and Hf_12_(precip) align, two distinct differences were observed: a 9.0 kDa fragment unique to Hf_12_(sol) and a 6.4 kDa fragment unique to Hf_12_(precip). The 9.0 kDa fragment likely arises from cleavage at multiple aspartate residues and, due to the lower hydrolysis efficiency of Hf_12_(precip) compared to Hf_12_(sol), its concentration may be insufficient to detect by SDS‐PAGE. The absence of the 6.4 kDa fragment with Hf_12_(sol) is more complex and most likely relates to differences in solution pH induced by the catalysts (Figure 4C,D). Hf_12_(sol) lowers the pH to approx. 4.5 under the conditions employed in the Mb hydrolysis reactions, likely due to residual cocrystallized acetic acid. In contrast, addition of Hf_12_(precip) does not alter the pH of the reaction mixture, which remains neutral. The 6.4 kDa fragment is probably generated by cleavage at D61, located in a positively charged region near the heme group. This positive charge is enhanced at pH 4.5 (Figure 4C), creating electrostatic repulsion with positively charged Hf_12_(sol) (as supported by zeta potential measurements, Figure S23), which further prevents interaction and cleavage. Conversely, at neutral pH, the reduced positive charge of the protein (from approx. +20 at pH 4.5 to 0 at pH 7.0) combined with the near‐neutral charge of the Hf_12_(precip) cluster (Figure S23) promotes interaction and facilitates coordination with D61 (Figure 4D). This example highlights the critical role of reaction medium pH in peptide bond hydrolysis, as it influences the charges of both the substrate and catalytic species.

To benchmark the Hf_12_ clusters, we compared their reactivity to structurally related metal‐oxo cluster catalysts. SDS‐PAGE shows that the selectivity is identical to that of the Zr_6_ dimer but cleavage proceeds more slowly, consistent with the reduced ligand exchange rate expected from the stronger Hf─O bonds [41]. Compared with other insoluble Hf‐oxo cluster systems (Hf_18_, Hf‐NU‐1000 MOF), Hf_12_(precip) exhibits markedly lower protein adsorption yet cleaves with a similar selectivity [19, 42]. Low adsorption, combined with high recyclability, renders Hf_12_(precip) advantageous for proteomics applications.

## 4 Conclusion

We synthesized a novel hexanuclear cluster, Hf_6_O_4_(OH)_4_(CH_3_CO_2_)_7_Cl_4_^+^ (Hf_6_), which serves as a precursor to both Hf_12_(sol), obtained by incubation of Hf_6_ in water at 60°C, and Hf_12_(precip), formed upon raising the pH of a Hf_6_ solution above 8.0. Both Hf_12_ species share identical core structures and have highly similar ligand shells that differ only in ligand occupancy rather than identity. However, this subtle difference governs the clusters’ solubility and ultimately has a substantial influence on their catalytic activity. This unprecedented structural similarity between metal‐oxo cluster‐based catalysts in two phases enabled a direct comparison of their catalytic behavior in peptide bond hydrolysis based solely on their phases, minimizing the influence of confounding factors. Notably, differences in the number of bidentate acetate ligands, which bind more strongly to the cluster core than monodentate chloride, hydroxide, or water ligands, substantially impact the catalytic activity. This was demonstrated by the superior hydrolysis efficiency of Hf_12_(precip) toward the dipeptide glycylglycine despite its heterogeneous nature, attributed to the loss of ±49% of acetate ligands. In addition, Hf_12_(precip) retains its insolubility across a broad pH range (2.0–12.0), enabling its reuse under varied conditions without compromising phase stability and with minimal loss of reactivity, thereby offering enhanced practical utility over its soluble counterpart. This is particularly important for proteomics where robust and recyclable artificial proteases are highly desired. On the other hand, the solubility of Hf_12_(sol) allows for detailed molecular‐level characterization of the reaction mechanism in solution, including analysis of coordination modes and the cluster‐induced structural changes of myoglobin. Both clusters exhibit identical cleavage selectivity, however, slight differences in resulting fragments can be attributed to variation in the electrostatic interactions between the cluster and protein, which are influenced by the pH. The observed correlations between the distinct phases of the clusters and their reactivity offer valuable mechanistic insight to inform the rational design of next‐generation catalysts with improved performance.

## Funding

Research Foundation Flanders (FWO) (G025624N, I002720N, 1267623N, 1253824N, G0D5419N), Swiss National Science Foundation (218106), KU Leuven (C14/23/088, CELSA/24/007, STG‐18‐00289, AKUL/19/45), Hercules Foundation (20100225–7).

## Conflicts of Interest

The authors declare no conflicts of interest.

## Supporting information




**Supporting File 1**: advs73508‐sup‐0001‐SuppMat.pdf.

**Supporting File 2**: advs73508‐sup‐0002‐Data.zip.

## Data Availability

The data that support the findings of this study are available in the supplementary material of this article.
