# Supplementary material for: From Solution to Surface: How the Catalytic Environment Modulates Peptide Bond Cleavage by Metal‐Oxo Cluster Nanozymes
Source: Adv Sci (Weinh). 2026 Jan 5;13(13):e19545. doi: 10.1002/advs.202519545 (PMC12955937; doi:10.1002/advs.202519545)
Supplement: Supplementary file 1 — Supporting File 1: advs73508‐sup‐0001‐SuppMat.pdf. [file ADVS-13-e19545-s002.pdf]

SUPPLEMENTARY INFORMATION – From Solution to Surface: How the Catalytic Environment Modulates Peptide Bond Cleavage by Metal-Oxo Cluster Nanozymes

Kilian Declerck[1], Muhammed Jibin Parammal[2], Carlotta Seno[2], Thomas J. N. Hooper[3], Dimitrios Sakellariou[3], Jonathan De Roo[2*], Nada D. Savić[1,4*], and Tatjana N. Parac-Vogt[1*]

[1] Department of Chemistry, KU Leuven, 3001 Leuven, Belgium

[2] Department of Chemistry, University of Basel, 4058 Basel, Switzerland

[3] Centre for Membrane Separations, Adsorption, Catalysis and Spectroscopy for Sustainable Solutions (cMACS), KU Leuven, 3001 Leuven, Belgium

[4] Institut Lavoisier de Versailles, CNRS, UVSQ, Université Paris-Saclay, 78035 Versailles, France

[*]Corresponding authors: tatjana.vogt@kuleuven.be; nada.savic@uvsq.fr, jonathan.deroo@unibas.ch

Supplementary Information

Figure	Description	Page
S1	(A) PXRD pattern of Hf_6, which is nearly identical to the one reported by Dai et al. for $Zr_6O_4(OH)_4(CH_3CO_2)_8(H_2O)_2Cl_3^+$ (Zr_6). The simulated PXRD pattern of the analogous $Zr_6O_4(OH)_4(CH_3CO_2)_{12}$ (Zr_6-acetate) cluster is included as a reference. (B) FT-IR spectra of the Hf_6 and Zr_6 cluster.	11
S2	PDF fit of the Hf_6 cluster with a Hf_6 model (CCDC 1051013), limited to 8 Å due to the material's high crystallinity.	12
S3	HR-MS of Hf_6 immediately after dissolution in 1:1 methanol:acetic acid (1 %) with $Hf_6O_4(OH)_4(CH_3CO_2)_7Cl_4^+$ at m/z 1755.80.	12
S4	(A) ^{1}H NMR spectrum of 2.0 mM Hf_6 and (B) ^{13}C NMR spectrum of 200 mM Hf_6 incubated in D_2O at 60 °C. (C) Deconvolution of the peaks at ~ 2.05 ppm corresponding to Hf-coordinated acetate (46.08%) and acetic acid (53.92 %) in the ^{1}H NMR spectrum of Hf_{12}(sol) (24 h, 60 °C).	13
S5	Diffusion ordered spectroscopy data of 5 mM Hf_6 (A) immediately after dissolution in D_2O or (B) after 24 h at 60 °C. Intensity decay curve fitted using a modified Stejskal-Tanner equation with a bi-exponential fit function (C) immediately after dissolution in D_2O or (D) after 24 h at 60 °C.	14
S6	Raman spectra of Hf_{12}(precip), $HfCl_4$- and $HfOCl_2$-based precipitates, and HfO_2.	15
S7	TEM images of Hf_{12}(precip) obtained after drop-casting dilute solutions of cluster in water. Based on measurements of 100 individual particles at the highest magnification, the average diameter was determined to be 4.93 ± 0.52 nm.	16
S8	^{1}H NMR spectra of the supernatant after precipitation of Hf_{12}(precip) from a 20.0 mM Hf_6 solution at pH 8.0 – 12.0, containing 1.0 mM TMSp-d_4.	17
S9	EDS images confirming the presence of both Na^+ and Cl^- ions.	18
S10	(A) Absorption spectra of 0.8 mM Hf_{12}(sol) and 0.8 μmol Hf_{12}(precip) after digestion using 1.0 mL of 1.0 M NH_4HCO_3 and addition of reagents used to perform the chloride test. (B) Linear calibration curve of chloride concentration versus absorbance at 500 nm (A_{500}).	18
S11	Thermogravimetric analysis of Hf_6 and Hf_{12}(precip) solids between 35 to 600 °C at a heating rate of 4 °C/min.	19
S12	^{1}H NMR spectrum of 0.8 μmol Hf_{12}(precip) after digestion using 1.0 mL of 1.0 M NH_4HCO_3 with 2.0 mM TMSp-d_4 as internal reference.	19
S13	^{1}H NMR spectra of 2.0 mM GG hydrolysis by an equimolar concentration of (A) Hf_{12}(sol) and (B) Hf_{12}(precip). First-order kinetics of the turnover of GG to G at 60 °C and pD 7.4 by (C) Hf_{12}(sol) and (D) Hf_{12}(precip).	20
S14	^{1}H NMR spectrum of supernatant after incubation of 2.0 μmol Hf_{12}(precip) in 1.0 mL D_2O at 60 °C for 24 h.	21

S15	Adsorption of 2.0 mM GG on 2.0 μmol Hf_{12}(precip) in 1.0 mL D_2O at 25 °C.	21
S16	Concentration of (A) GG and (B) G in function of reaction time. Based on 0th order kinetics, GG disappears with a rate constant of 9.64×10^{-6} s^{-1} whereas G appears with a rate constant of 7.15×10^{-6} s^{-1}.	22
S17	Conversion of 2.0 mM GG to G after 24 h at 60 °C using the precipitates derived from 24.0 mM $HfCl_4$ and $HfOCl_2$, compared to conversion caused by 2.0 μmol Hf_{12}(precip) in 1.0 mL D_2O.	22
S18	PDF fits of Hf_{12}(precip) and precipitates derived from $HfCl_4$ and $HfOCl_2$ with an oxygen-bridged Hf_{12} model structure extracted from CCDC 2002902.	23
S19	Absorption spectrum of 0.01 mM Mb in the presence and absence of Hf_{12}(sol).	23
S20	Fluorescence emission spectrum of 0.01 mM Mb in the presence and absence of Hf_{12}(sol).	24
S21	CD spectrum of 0.01 mM Mb in the presence and absence of Hf_{12}(sol).	24
S22	Adsorption of 0.02 mM Mb on 1.0 – 2.0 μmol Hf_{12}(precip) in function of incubation time at 25 °C in 1.0 mL of water.	25
S23	Zeta potential of 2.0 mM Hf_{12}(sol) and 2.0 μmol Hf_{12}(precip) at their native pH in 1.0 mL of water.	25

Table	Description	Page
S1	The Hf(IV) concentration and corresponding % Hf(IV) as fraction of the initial amount of Hf_6 after precipitation of 1.0 mM Hf_{12}(sol) by addition of 1.0 M NaOH to reach pH 8.0 – 12.0.	26
S2	The Hf(IV) concentration and corresponding % Hf(IV) as fraction of the initial amount of Hf_{12}(precip) after incubation of 1.0 μmol Hf_{12}(precip) in 1.0 mL of water at 60 °C for 144 h at pH 2.0 – 12.0.	26
S3	Refinement parameters for all PDF fits.	26
S4	Hydrolysis efficiency across reaction conditions with Hf_{12}(sol) and Hf_{12}(precip) and the relative abundance of individual fragments generated by both clusters (%).	27
S5	The fragments observed by SDS-PAGE after cleavage of Mb (in kDa) using Hf_{12}(sol) or Hf_{12}(precip) can be attributed to aspartate selective cleavage according to the comparison with fragments obtained for the Zr_6 dimer that were analyzed using LC-MS/MS.	27

Experimental Procedures

Materials

Acetic acid (99.9 %), acetone, ammonium bicarbonate, bromophenol blue, deuterium oxide, deuterium chloride (35 wt%), ethanol, ethylene glycol (99 %), equine skeletal muscle myoglobin (95+ %, Mb), formaldehyde, glutaraldehyde, hydrochloric acid (37 %), isopropanol, silver nitrate, sodium deuteroxide (30 wt%), sodium dodecyl sulfate (SDS), *N,N,N',N'*-tetramethyl ethylenediamine (TEMED), 3-(trimethylsilyl)propionic-2,2,3,3-d_4 acid sodium salt (TMSp-d_4), and hafnium chloride were acquired from Sigma-Aldrich. Ammonium persulfate, dithiothreitol (> 97 %), ethanol, glycerol, glycylglycine (99 %), methanol, PageRuler™ unstained low range protein ladder, sodium carbonate, sodium thiosulfate, tricine, and tris-(hydroxymethyl)aminomethane were purchased from Thermo Fisher Scientific. Acrylamide/bis-acrylamide (40 %) was purchased from PanReact Applichem. All chemicals and substrates were used without further purification.

Synthesis and Characterization of Hf_6

The $Hf_6O_4(OH)_4(CH_3CO_2)_7Cl_4^+$ (Hf_6) cluster was synthesized according to the published procedure for the $Zr_6O_4(OH)_4(CH_3CO_2)_8(H_2O)_2Cl_3^+$ (Zr_6) cluster using hafnium chloride instead of zirconium chloride.[1] More specifically, $HfCl_4$ (6.88 g, 21.5 mmol, 1 equiv.) and acetic acid (7.80 g, 130 mmol, 6 equiv.) were added to 12.5 mL isopropanol under stirring at 500 rpm at 120 °C for 1.5 h. The product was collected through suction filtration and subsequently washed twice with acetone and dried under vacuum at 25 °C. The Hf_6 cluster was analyzed using Fourier transform infrared (FT-IR) and Raman spectroscopy, powder X-ray diffraction (PXRD), pair distribution function (PDF) analysis of total X-ray scattering, and high-resolution mass spectrometry (HR-MS). FT-IR spectra were collected using a Bruker Vertex 70 FT-IR spectrometer. Raman spectra were measured on a Bruker Vertex 70 FT-IR spectrometer coupled to a RAM II FT-Raman module equipped with a Nd:YAG laser source (1064 nm) at 200 mW. PXRD patterns were collected on a Malvern PANalytical Empyrean diffractometer (in transmission mode) over a 1.3 – 45° 2θ range, using a PIXcel3D solid-state hybrid pixel detector and Cu anode (Cu Kα1: 1.5406Å; Cu Kα2: 1.5444Å). X-ray total scattering data were collected at beamline P21.1 at PETRA III/DESY in Hamburg, Germany. High-resolution mass spectra were acquired on a quadrupole orthogonal acceleration time-of-flight mass spectrometer (Synapt G2 HDMS, Waters, Milford, MA). Samples were dissolved in 50:50 methanol:acetic acid (1 %) and infused at 3.0 µL/min. HR-MS spectra were obtained in positive ionization mode with a resolution of 15000 (FWHM) using leucine enkephalin as lock mass.

Characterization and Stability of Hf_{12}(sol) and Hf_{12}(precip)

Hf_{12}(sol) was formed by incubation of Hf_6 in water at 60 °C for at least 3 h, while Hf_{12}(precip) was formed by adding 1.0 M NaOH to a solution containing Hf_6 or Hf_{12}(sol) to reach pH > 8. Both materials were characterized immediately after their formation, either in solution or as solids, by FT-IR and Raman spectroscopy, PDF analysis, transmission electron microscopy paired with energy dispersive X-ray spectroscopy (TEM-EDS), a Spectroquant chloride test, solution- and solid-state nuclear magnetic resonance (NMR) spectroscopy, thermogravimetric analysis (TGA), and inductively coupled plasma optical emission spectroscopy (ICP-OES). Stability was assessed by incubating 2.0 – 10.0 mM Hf_{12}(sol) or 2.0 – 10.0 µmol Hf_{12}(precip) in 1.0 mL of water at 60 °C for up to 144 h with the techniques described above.

FT-IR and Raman Spectroscopy

Both FT-IR and Raman spectra of Hf_{12}(precip) immediately after precipitation were collected. The FT-IR spectra were recorded using a Bruker Vertex 70 FT-IR spectrometer. Raman spectra were recorded on a Bruker Vertex 70 FT-IR spectrometer coupled to the RAM II FT-Raman module, equipped with a 1064 nm Nd:YAG laser operating at 200 mW.

PDF Analysis

X-ray total scattering data, from which PDFs were obtained, were collected at beamline P21.1 at PETRA III/DESY in Hamburg, Germany. Samples were measured using a PerkinElmer digital X-ray flat-panel amorphous silicon detector with a resolution of 2048 × 2048 pixels and a pixel size of 200 × 200 µm, positioned at a sample-to-detector distance of 365 mm. The incident X-ray wavelength was 0.10154 Å, and each measurement was recorded with an exposure time of 600 seconds. Solid and liquid (10 mM Hf_{12}(sol)) samples were loaded into 2 mm polyamide Kapton tubes and measured at room temperature in rapid acquisition mode. Instrument calibration was performed using a silicon standard. Data integration was conducted with the pyFAI software[2] package. PDFs were generated using xPDFSuite and PDFgetX3[3] with Q_{max} = 17.5 Å, Q_{min} = 0.8 Å, and R_{poly} = 0.9. PDF refinements were carried out using Diffpy-CMI[4] by refining the scale factor, isotropic atomic displacement parameters (Uiso), delta2 (accounting for $1/r^2$ peak sharpening), and zoom factor (**Table S3**). Specific cluster models used for PDF fitting are provided as xyz files.

Transmission Electron Microscopy paired with Energy Dispersive X-ray Spectroscopy

Hf_{12}(precip) was suspended in water and deposited on a Holey Carbon – Cu, 300 mesh, 50-micron grid from EMS. TEM was conducted using a JEOL JEM-1400Flash instrument operating at 120 kV. EDS mapping was conducted by using an Ultim Max EDS detector integrated into the same JEOL JEM-1400Flash microscope. TEM images were analyzed using ImageJ.

Solution-State NMR Spectroscopy

Solution-state ^{1}H and ^{13}C NMR spectroscopy was used to monitor the acetic acid release from the clusters after incubation of 2.0 – 200 mM Hf_{12}(sol) or 2.0 – 200 µmol Hf_{12}(precip) in 1.0 mL D_2O at 60 °C. In addition, the ^{1}H NMR spectrum of 0.8 µmol Hf_{12}(precip) digested with 1.0 mL of 1.0 M NH_4HCO_3 for 1 h under sonication was measured. Spectra were recorded on a Bruker Avance III HD 400 MHz spectrometer using standard 5 mm NMR tubes and 2.5 mM TMSp-*d₄* as an internal reference. A single-pulse sequence at a 30° flip angle (3.4 µs pulse duration, 1 s delay, 3.3 s acquisition time) was used to acquire the ^{1}H NMR, whereas ^{13}C NMR spectra were recorded using a pulse sequence with power-gated decoupling and a 30° flip angle (2.6 µs pulse duration, 2 s delay, 1.4 s acquisition time). Data processing was performed using ACD/Labs® Spectrus Processor™. Diffusion ordered spectroscopy (DOSY) measurements were performed using a double stimulated echo sequence with bipolar gradient pulses (dstebpgp3s). The gradient pulse duration and diffusion delay were adjusted to achieve a final signal attenuation of <10 % in the last increment relative to the first. Diffusion coefficients were extracted by fitting the signal intensity decay with a modified Stejskal-Tanner equation:

$$I = I_0 e^{-(\gamma\delta g)^2 D(\Delta - 0.6\delta)}$$

where I denotes the signal intensities, D the linear diffusion coefficients, γ the gyromagnetic ratio of the observed nucleus, g the gradient strength, δ the pulsed-field gradient duration (0.0015), and Δ the diffusion delay (0.35). A correction factor of 0.6 was applied to δ to account for the smoothed squared gradient pulse shape.

Solid-State NMR Spectroscopy

Solid-state ^{1}H and ^{13}C NMR spectroscopy was used to monitor changes in the coordinated acetate ligands on Hf_{12}(precip) after incubation of 2.0 μmol Hf_{12}(precip) in 1.0 mL D_2O at 60 °C for 24 h in the presence and absence of GG. Measurements were performed on an 11.7 T wide-bore Oxford Instruments electromagnet with a Bruker Avance NMR spectrometer and a 2.5 mm Chemagnetics HXY MAS probe, allowing a MAS frequency of 12 kHz to be used for all of the experiments. All spectra were processed using the Topspin software package and referenced to the unified scale using IUPAC recommended frequency ratios relative to the ^{13}C methylene resonance of adamantane (δ = 37.77 ppm).[5-6] Spectral deconvolution and integration was performed with dmfit.[7] The ^{1}H NMR spectra were collected with a one-pulse sequence at v_0 = 499.80 MHz (4 scans, 2.8 μs 90° pulse, 4.5 – 20 s relaxation delays). The ^{13}C NMR spectra were collected with a CPMAS pulse sequence at v_0 = 125.68 MHz (4000 scans, 5000 μs contact pulse, 1 – 10 s relaxation delays, high-power proton decoupling). Optimal relaxation delays were determined by ^{1}H saturation recovery experiments, which utilized a 200-pulse saturation pulse train.

Chloride Test

Samples containing 0.8 mM Hf_{12}(sol) or 0.8 μmol Hf_{12}(precip) were incubated in 1.0 mL of 1.0 M NH_4HCO_3 at 60 °C for 24 h to ensure complete digestion.[8] Quantitative analysis of chloride ions in solution was performed using the Spectroquant chloride test kit from Merck. The test is suitable for concentrations ranging from 10 to 250 mg/L, so a calibration series across this range was also measured. In this assay, chloride ions react with mercury(II) thiocyanate to form mercury(II) chloride, releasing thiocyanate ions that can subsequently react with iron(III) ions to produce red iron(III) thiocyanate, which is quantified photometrically at A_{500} using the Tecan Spark® Multimode Microplate Reader.

Thermogravimetric Analysis

TGA was performed for 10 mg of Hf_6 or Hf_{12}(precip) using a NETZSCH STA 449 F3 thermal analyzer. The temperature was ramped from 35 °C to 600 °C at a heating rate of 4 °C/min.

Inductively Coupled Plasma Optical Emission Spectroscopy

Precipitation of 2.0 mM Hf_{12}(sol) was induced by adjusting pH to 8 – 12 using 1.0 M NaOH, followed by collection of the supernatant. Additionally, solutions of 2.0 μmol Hf_{12}(precip) in 1.0 mL of water were incubated at 60 °C for 144 h across a pH range of 2 – 12, and the resulting supernatant was collected. ICP-OES measurements were performed using a PerkinElmer Optima 8300 instrument operated in axial view. Supernatant samples were diluted 10-fold in 2 % HNO_3 prepared in MilliQ water, and Hf concentrations were quantified using a standard calibration curve ranging from 0.001 to 50 ppm.

Zeta Potential Measurements

Zeta potential measurements of 2.0 mM Hf_{12}(sol) or 2.0 μmol Hf_{12}(precip) in 1.0 mL of water were performed using DTS1070 disposable folded capillary cells on the Malvern PANalytical ZetaSizer Nano ZSP. The electrophoretic mobility was determined via laser Doppler velocimetry, and the corresponding zeta potentials were calculated using the Smoluchowski approximation in ZetaSizer 7.12. For the protein myoglobin (Mb), a charge of +20 and 0 were calculated at pH 4.5 and 7.0, respectively, using the Prot Pi protein tool.[9]

Elemental Analysis

C, H, and N contents were measured by quantitative elemental analysis using a Vario MICRO cube from Elementar. The Hf and Cl contents were obtained from TGA and the chloride test, respectively. For the Hf_6 cluster, the measured values (%) were Hf 55.77, Cl 6.73, C 9.97, and H 2.54 (calculated: Hf 60.92, Cl 8.07, C 9.06, H 1.43). The discrepancy is attributed to residual trapped water (± 6.46 wt%) and isopropanol from synthesis (± 2.19 wt%). For Hf_{12}(precip), the measured values were Hf 57.92, Cl 4.43, C 4.32, and H 1.97 (calculated: Hf 63.80, Cl 4.96, C 3.94, H 1.90). This discrepancy is attributed to additional water (± 2.43 wt%) and Na species, which were confirmed by EDS, formed during precipitation (most likely ± 6.79 wt% Na_2CO_3). The solvent content in both clusters is consistent with >10 % mass loss below 200 °C in TGA.

Dipeptide Experiments

Hydrolysis of Glycylglycine

Solutions containing 2.0 mM glycylglycine (GG) and 2.0 mM Hf_{12}(sol) or 2.0 μmol Hf_{12}(precip) were prepared in 1.0 mL D_2O, and the pD was adjusted to 7.4 using DCl and NaOD before incubation at 60 °C for 48 h. pH-meter readings were corrected according to the equation: pD = pH + 0.41. ^{1}H NMR spectra were recorded on a Bruker Avance 400 MHz spectrometer using 2.5 mM TMSp-*d₄* as an internal reference. Spectral analysis was performed using ACD/Labs® Spectrus Processor™. The decrease of the GG concentration over time was fitted using a mono-exponential decay function to determine the observed rate constant and the corresponding half-life of hydrolysis. Conversion of GG was also monitored for precipitates derived from 24.0 mM $HfCl_4$ and $HfOCl_2$ at 60 °C. Finally, adsorption of GG was evaluated for Hf_{12}(precip) (2.0 μmol in 1.0 mL D_2O) under milder conditions (8 h, 25 °C) to prevent hydrolysis.

Interaction between Hf_{12}(sol) and GG

Solutions of 2.0 mM Hf_{12}(sol) and 2.0 – 48.0 mM GG were incubated for 2 h at 60 °C and pD 5.0 to study the interactions and avoid hydrolysis. ^{1}H NMR spectra were recorded on a Bruker Avance 400 MHz spectrometer using 2.5 mM TMSp-d_4 as an internal reference. Spectral analysis was performed using ACD/Labs® Spectrus Processor™.

Recycling of Hf_{12}(precip) Catalyst

The recyclability of Hf_{12}(precip) in GG hydrolysis was evaluated over four consecutive reaction cycles (as described above) at pD 7.4 and 60 °C. The cluster was recovered by centrifugation after each cycle and washed three times with water (two times for 1 h and one extended wash of 24 h) to remove unreacted substrate and any adsorbed hydrolysis products. Complete removal of GG and G from Hf_{12}(precip) was confirmed by ^{1}H NMR analysis, which showed no detectable signals corresponding to the substrate or product. The solid was then air-dried and reused under identical reaction conditions for subsequent hydrolysis cycles.

Protein Experiments

Protein Hydrolysis Studies

Aqueous solutions containing 0.02 mM Mb and 0.04 – 0.2 mM Hf_{12}(sol) (pH 4.5) or 0.1 – 2.0 μmol Hf_{12}(precip) (pH 7.4) were prepared in 1.0 mL of water without pH adjustment. Control experiments were conducted under identical conditions in the absence of clusters. Aliquots were taken at different time intervals, and for Hf_{12}(precip)-containing samples, the cluster was

removed by centrifugation prior to analysis. Reaction progress was monitored by sodium dodecyl sulfate-polyacrylamide gel electrophoresis (SDS-PAGE). For each sample, 15 µL was mixed with 5 µL of loading buffer, heated to 95 °C for 5 minutes, and centrifuged for 3 minutes. A 10 µL aliquot was loaded onto gels consisting of a 5 % stacking layer and an 18 % resolving layer. A PageRuler™ Low Range Unstained Protein Ladder (3.4 – 100 kDa) was used as molecular weight marker. Electrophoresis was conducted for 2 h at 200 V using an OmniPage system powered by a Consort EV243 power supply, with a constant current of 35 mA/gel and maximum power output of 50 W. Tris-Tricine buffer containing 0.1 % SDS was used as running buffer. After electrophoresis, gels were silver-stained to visualize bands belonging to protein and generated peptides. Imaging and analysis were performed using a GelDoc™ EZ system and Image Lab software (Bio-Rad).

Protein Interaction with Hf_{12}(sol)

The effect of 0 – 10 equiv. or 0 – 0.1 mM Hf_{12}(sol) on 0.01 mM Mb was characterized using UV-Vis, fluorescence, and circular dichroism spectroscopy. UV-Vis absorption spectra were acquired in single-beam mode on an Agilent Cary 6000i spectrophotometer using a 1 cm quartz cuvette over a wavelength range of 200 – 800 nm. Spectral changes were monitored in the Soret band (409 nm) and Q-band region (500 – 600 nm), both characteristic of the heme group. Baseline correction was performed by subtracting the spectrum of water. Fluorescence measurements, which probe the environment of tryptophan residues, were performed in triplicate at 25 °C on an Edinburgh Instruments FLS-980 spectrofluorometer equipped with a xenon lamp (230 – 1000 nm). Excitation was set at 286 nm, and emission was recorded from 300 to 440 nm. Circular dichroism spectra were collected at 25 °C using 1 mm path length cuvettes on a JASCO J-1500 spectrometer, with the final spectrum obtained as an average of 3 scans and corrected against the water background. As previously demonstrated in similar studies, where acetic acid was used to mimic the cluster-induced acidity, observed spectral changes were primarily attributed to interaction with Hf_{12}(sol) rather than acidification of the medium.[10]

Protein Adsorption on Hf_{12}(precip)

Solutions containing 0.02 mM Mb and 1.0 – 2.0 µmol Hf_{12}(precip) were incubated in 1.0 mL of water at 25 °C for up to 12 h, and aliquots were collected at defined time points. Protein concentrations were quantified using the Bradford assay. For each measurement, 30 µL of sample was mixed with 270 µL of Bradford reagent in a clear 96-well microplate, shaken for 30 s, and incubated at 25 °C for 10 minutes. Absorbance spectra were recorded using a Tecan Spark® Multimode Microplate Reader from 400 to 650 nm in 5 nm increments, allowing detection of both the unbound dye (450 nm) and the protein-dye complex (590 nm). A calibration curve using 0 – 0.02 mM Mb was used to linearize the data.[11]

Statistical Analysis

Quantitative data were calculated as mean values derived from triplicate (n = 3) experimental measurements to ensure reproducibility. Error bars, representing the standard deviation, were added where necessary. No data preprocessing (e.g., transformation, normalization, or outlier removal) was applied. The only exception was the normalization of FT-IR and Raman spectra, which were used solely for qualitative comparison. This was noted in the corresponding figure captions.

Calculation of the Minimal Formula

The minimal formula for Hf_{12}(precip) was calculated according to *Pulparayil Mathew et al.*,[8] who provide a detailed explanation of the procedure. We start by writing the minimal formula of Hf_{12}(precip) as $Hf_{12}(\mu_3\text{-}O)_8(\mu_3\text{-}OH)_8(\mu_2\text{-}OH)_6(CH_3CO_2)_a(Cl)_b(OH)_c(H_2O)_d$.

Experimental Molar Mass from TGA

Complete thermal degradation of one mole of Hf_{12}(precip) results in the formation of 12 moles of HfO_2, accompanied by a mass loss of 31.69 % **(Figure S9)**.

$$12n_{Hf_{12}} = n_{HfO_2}$$

According to $n = W/M$, the number of moles is determined by dividing the mass of the cluster (W) by its molar mass (M). From the initial weight (W_I) and weight after combustion (W_{HfO_2}) in TGA, the experimental molar mass (M_{EXP}) can be obtained using:

$$M_{EXP} = \frac{W_I}{W_{HfO_2}} \times 12M_{HfO_2} = \frac{100\ \%}{68.31\ \%} \times 2525.88\ g/mol = 3697.67\ g/mol$$

Acetate Ligand Content from [1]*H NMR Spectroscopy*

Hf_{12}(precip) was digested using 1.0 mL of 1.0 M NH_4HCO_3 and ^{1}H NMR was acquired with 2.0 mM TMSP-d_4 as an internal reference **(Figure S10)**. With the integral of TMSP-d_4 normalized to 9, the concentration of acetate ligands is given by:

$$[CH_3CO_2^-] = \frac{I_{CH_3CO_2^-}}{3} \times [TMSp] = \frac{6.65}{3} \times 2.0\ mM = 4.43\ mM$$

At a Hf_{12}(precip) loading of 0.80 μmol, calculated using the experimental molar mass, the number of acetate ligands was calculated as:

$$a = \frac{[CH_3CO_2^-]}{[Hf_{12}(precip)]} = \frac{4.43\ mM}{0.80\ mM} = 5.5$$

Chloride Ligand Content from UV-Vis Spectroscopy

Hf_{12}(precip) was digested using 1.0 mL of 1.0 M NH_4HCO_3 and the Spectroquant chloride test was used to obtain the chloride concentration **(Figure S8)**. The final Hf_{12}(precip) loading was 0.80 μmol, calculated using the experimental molar mass.

$$b = \frac{[Cl]}{[Hf_{12}(precip)]} = \frac{3.76\ mM}{0.80\ mM} = 4.7$$

Structural and Electronic Balancing of the Hf_{12} Cluster

The minimal formula is charge-balanced by monodentate hydroxide ligands according to:

$$C^{Hf^{4+}} = C^{\mu_3-O^{2-}} + C^{\mu_3-OH^-} + C^{\mu_2-OH^-} + C^{CH_3CO_2^-} + C^{Cl^-} + C^{OH^-}$$

$$12 \times 4 = (8 \times 2) + 8 + 6 + 5.5 + 4 + c$$

$$c = 48 - 16 - 8 - 6 - 5.5 - 4.7 = 7.8$$

With acetate as bidentate and chloride and hydroxide as monodentate ligands, it is necessary to verify if the minimal formula provides coordinatively saturated Hf(IV) centers. Unsaturated Hf(IV) centers are most probably occupied by aqua ligands. Because the oxygen-bridged Hf_{12} cluster is fully saturated with 18 bidentate capping ligands, equal to 36 coordination bonds, the minimal formula can be written as follows:

$Hf_{12}(\mu_3\text{-}O)_8(\mu_3\text{-}OH)_8(\mu_2\text{-}OH)_6(CH_3CO_2)_{5.5}(Cl)_{4.7}(OH)_{7.8}(H_2O)_{12.5}$

Supplementary Figures

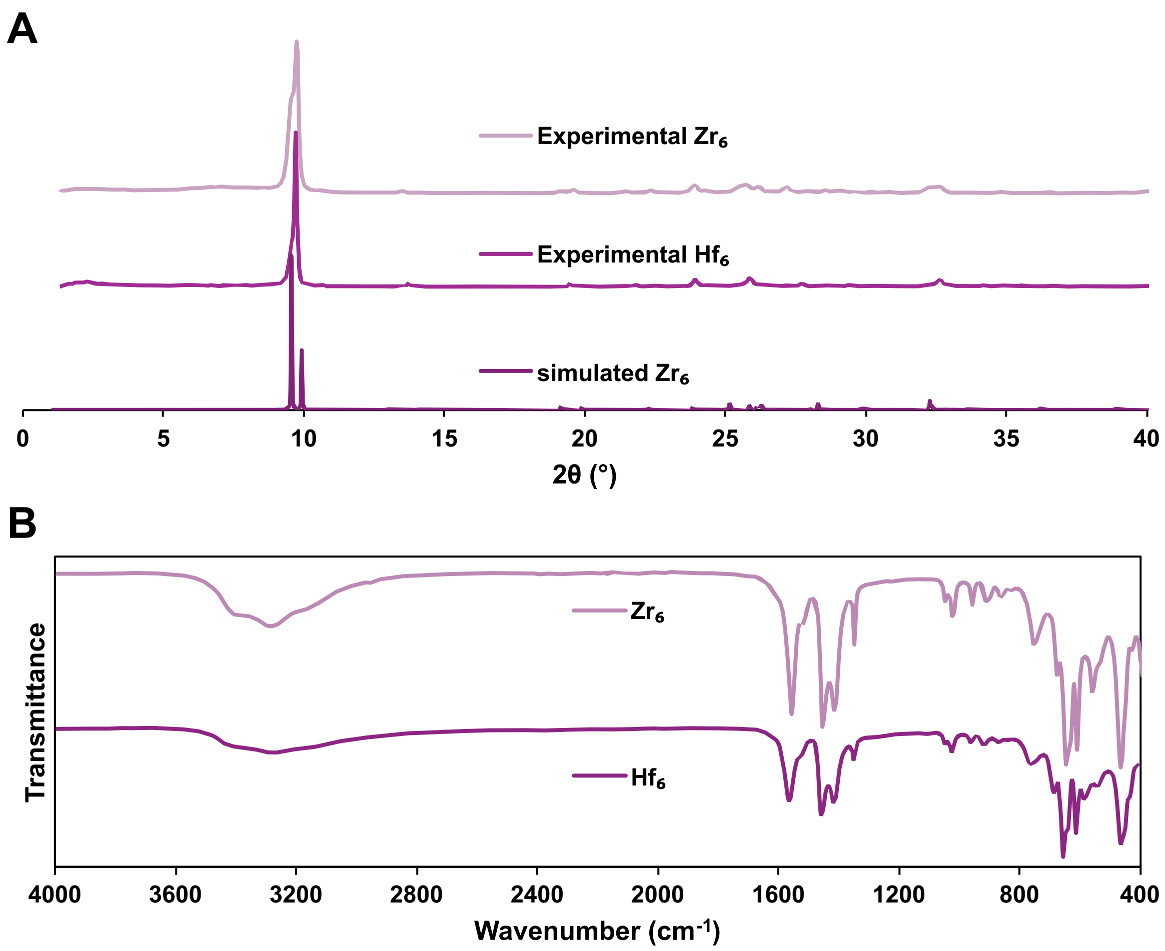

Figure S1 (A) PXRD pattern of Hf_6, which is nearly identical to the one reported by *Dai et al.* for $Zr_6O_4(OH)_4(CH_3CO_2)_8(H_2O)_2Cl_3^+$ (Zr_6).[1, 10] The simulated PXRD pattern of the analogous $Zr_6O_4(OH)_4(CH_3CO_2)_{12}$ (Zr_6-acetate) cluster is included as a reference.[12] (B) Normalized FT-IR spectra of the Hf_6 and Zr_6 cluster.

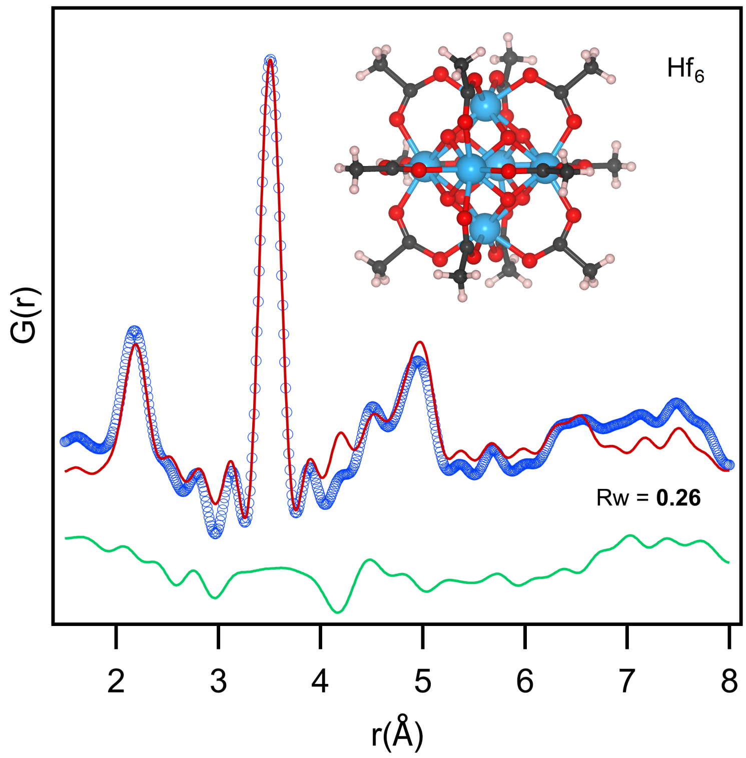

Figure S2 PDF fit of the Hf_6 cluster with a Hf_6 model (CCDC 1051013[12]), limited to 8 Å due to the high crystallinity (the model cif file is provided in the Supplementary Information

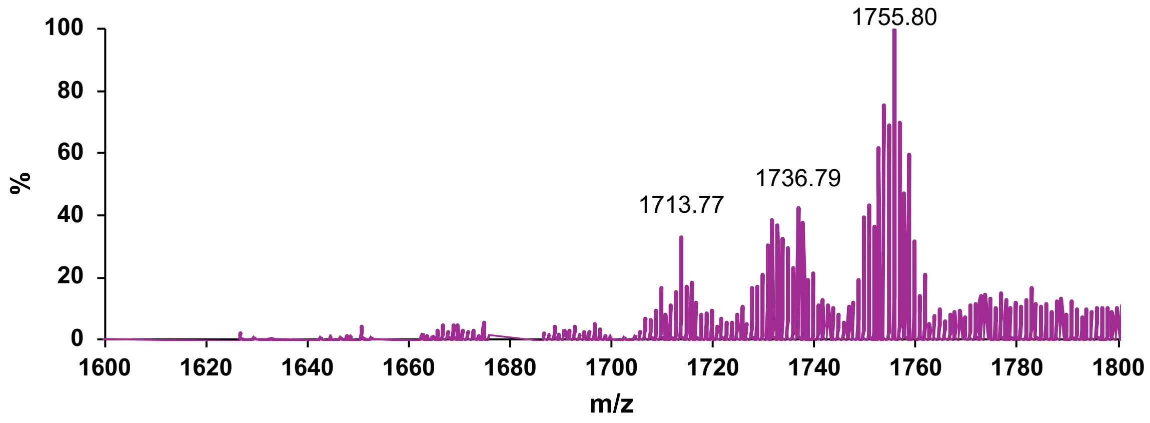

Figure S3 HR-MS of Hf_6 immediately after dissolution in 1:1 methanol:acetic acid (1 %) with $Hf_6O_4(OH)_4(CH_3CO_2)_7Cl_4^+$ at m/z 1755.80.

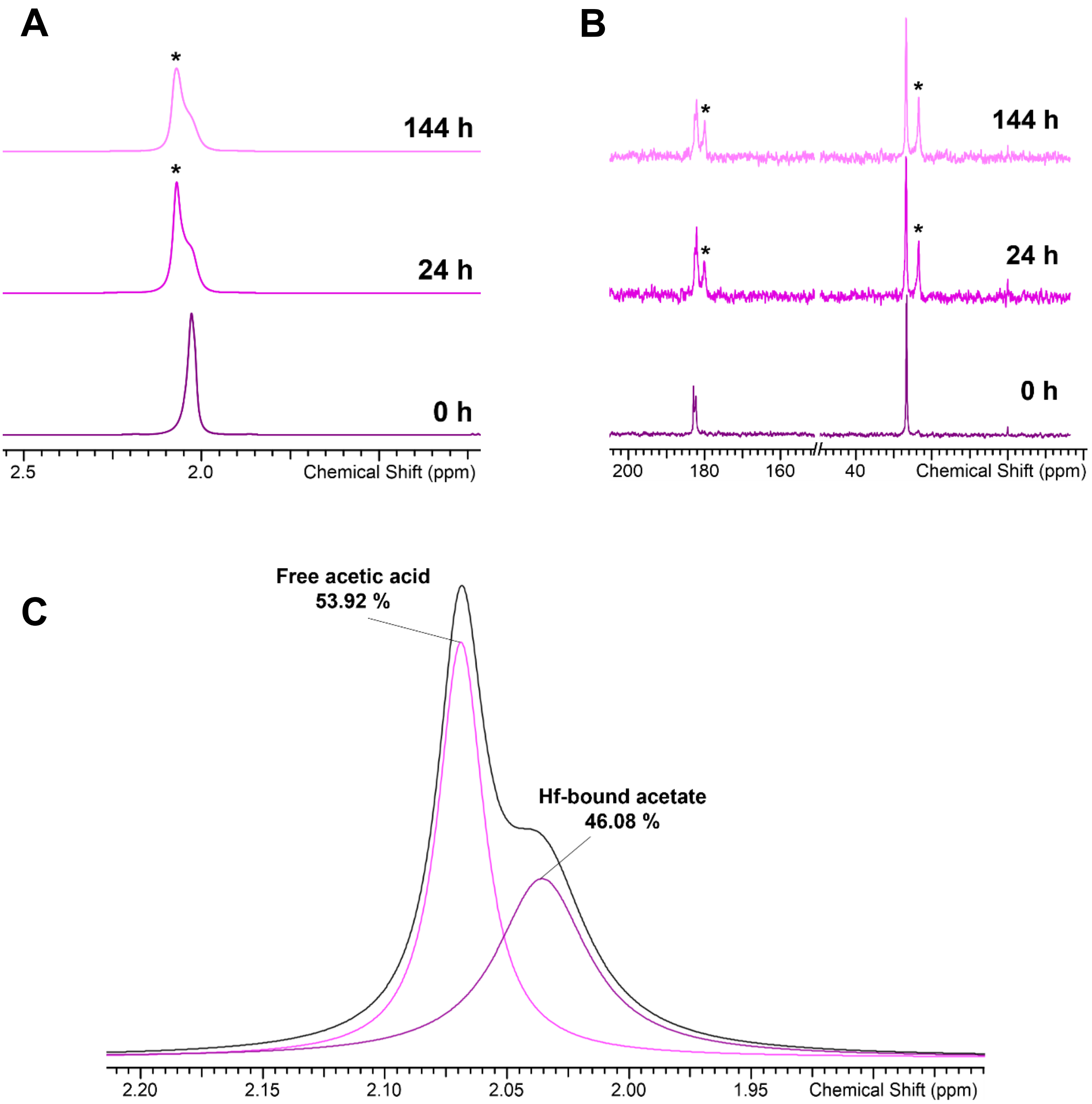

Figure S4 (A) ^{1}H NMR spectrum of 2.0 mM Hf_6 incubated in D_2O at 60 °C. (B) ^{13}C NMR spectrum of 200 mM Hf_6 incubated in D_2O at 60 °C. Released acetic acid after incubation is denoted by *. (C) Deconvolution of the peaks at ~ 2.05 ppm corresponding to Hf-coordinated acetate (46.08%) and acetic acid (53.92 %) in the ^{1}H NMR spectrum of Hf_{12}(sol) (24 h, 60 °C).

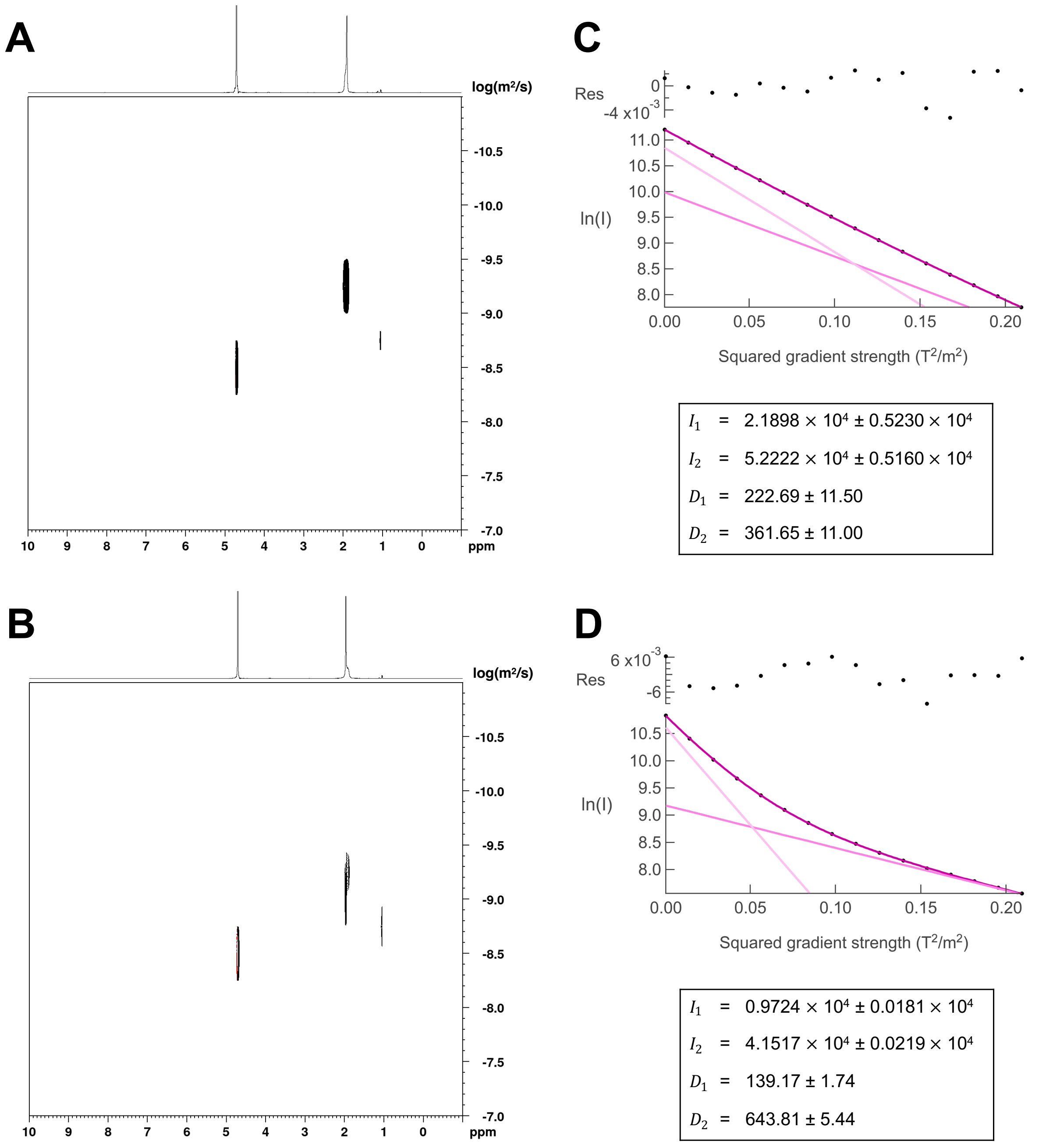

Figure S5 Diffusion ordered spectroscopy data of 5 mM Hf_6 (A) immediately after dissolution in D_2O or (B) after 24 h at 60 °C. Intensity decay curve fitted using a modified Stejskal-Tanner equation with a bi-exponential fit function (C) immediately after dissolution in D_2O or (D) after 24 h at 60 °C. I denotes signal intensities and D linear diffusion coefficients. Before incubation, the sample contains Hf_6 (361.65 μm^2/s, 1.35 nm) and a limited number of dimers that formed during the sample preparation (222.69 μm^2/s, 2.19 nm). After incubation, the sample contains Hf_{12}(sol) (139.17 μm^2/s, 3.51 nm) and free acetic acid (643.81 μm^2/s, 0.76 nm).

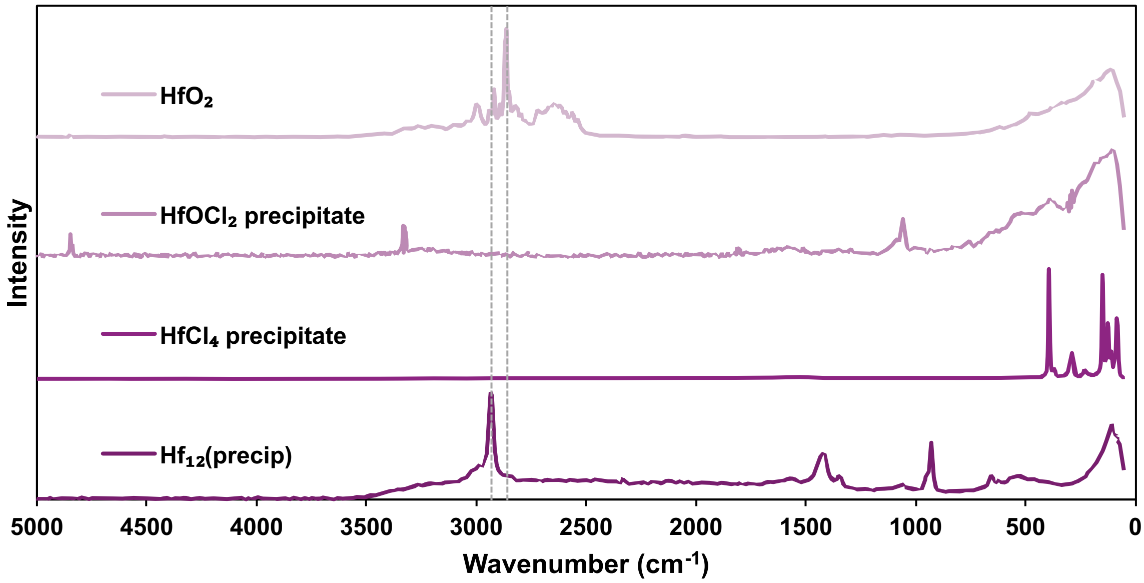

Figure S6 Normalized Raman spectra of Hf$_{12}$(precip), HfCl$_4$- and HfOCl$_2$-based precipitates (synthesized by dissolving HfCl$_4$ and HfOCl$_2$ in water and adding 1.0 M NaOH), and HfO$_2$.

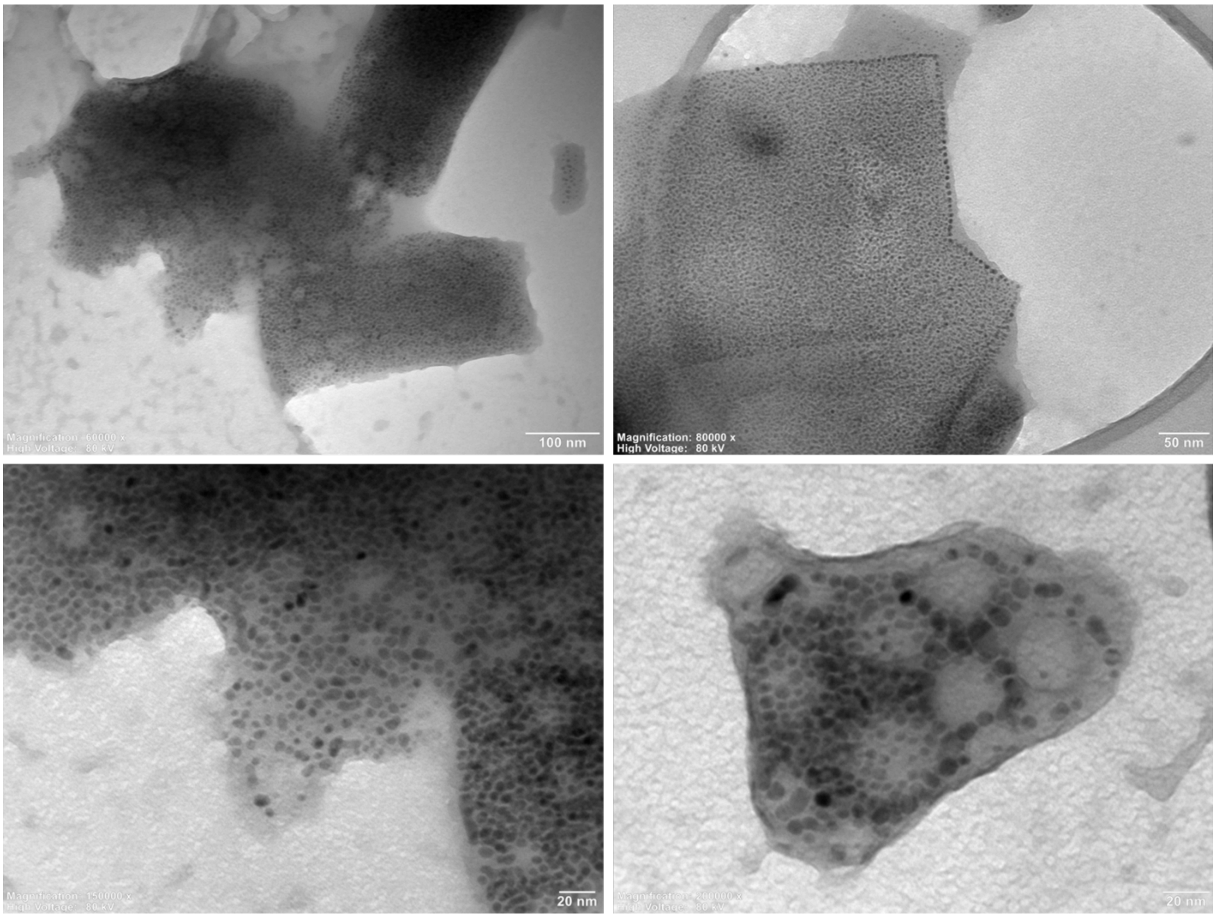

Figure S7 TEM images of Hf_{12}(precip) obtained after drop-casting dilute solutions of cluster in water onto a Holey Carbon – Cu TEM grid. Based on measurements of 100 individual particles at the highest magnification, the average diameter was determined to be 4.93 ± 0.52 nm.

$$[CH_3CO_2]_{pH\ 8.0} = [TMSp] \times \frac{I_{CH_3CO_2}/H_{CH_3CO_2}}{I_{TMSp}/H_{TMSp}} = 1\ mM \times \frac{18.00/3}{1.00/9} = 54\ mM$$

$$\frac{54\ mM}{(7 \times 20\ mM)} \times 100\ \% = 38.57\ \%\ at\ pH\ 8.0$$

$$[CH_3CO_2]_{pH\ 12.0} = 1\ mM \times \frac{22.70/3}{1.00/9} = 68.1\ mM$$

$$\frac{68.1\ mM}{(7 \times 20\ mM)} \times 100\ \% = 48.64\ \%\ at\ pH\ 12.0$$

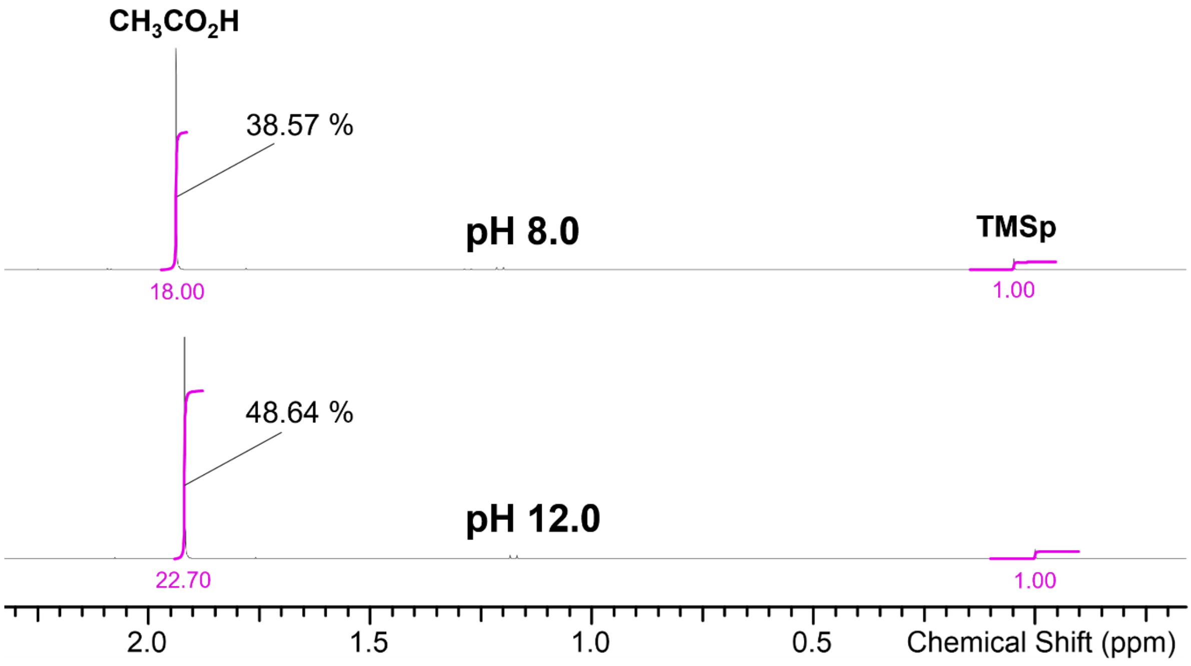

Figure S8 ^{1}H NMR spectra of the supernatant after precipitation of Hf_{12}(precip) from a 20.0 mM Hf_6 solution at pH 8.0 – 12.0, containing 1.0 mM TMSp-d_4.

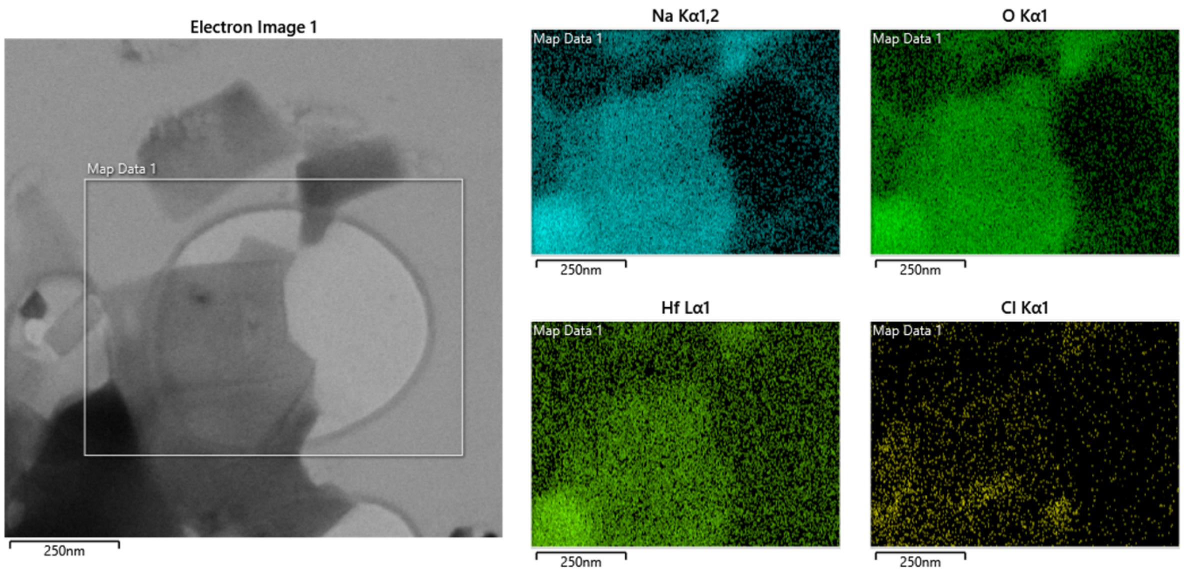

Figure S9 EDS images confirming the presence of both Na^+ and Cl^- ions.

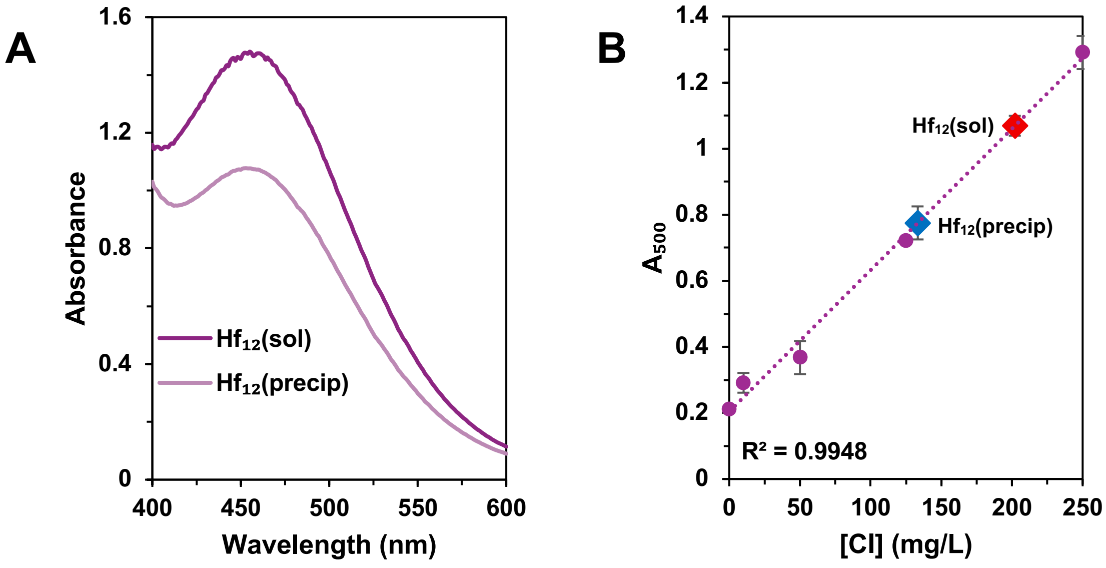

Figure S10 (A) Absorption spectra of 0.8 mM Hf_{12}(sol) and 0.8 μmol Hf_{12}(precip) after digestion using 1.0 mL of 1.0 M NH_4HCO_3 and addition of reagents used to perform the chloride test. (B) Linear calibration curve of chloride concentration versus absorbance at 500 nm (A_{500}) with the sample containing Hf_{12}(sol) in red (202.0 mg/L, 6.73 wt%) and Hf_{12}(precip) in blue (133.2 mg/L, 4.43 wt%). Measurements were performed in triplicate. Error bars represent the standard deviation.

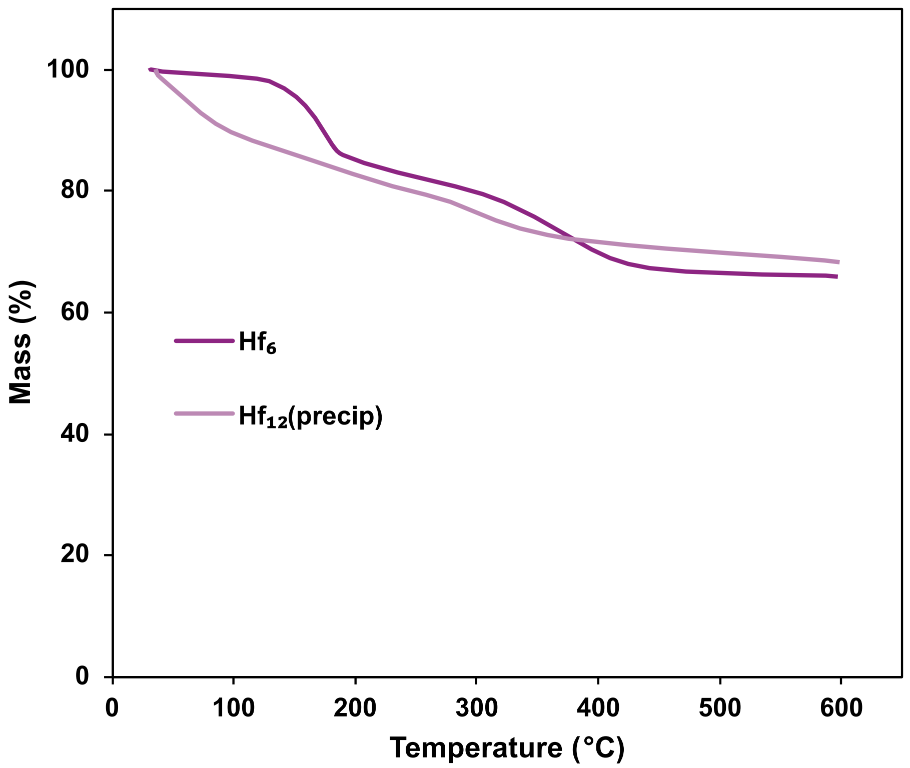

Figure S11 Thermogravimetric analysis of Hf_6 and Hf_{12}(precip) solids between 35 to 600 °C at a heating rate of 4 °C/min.

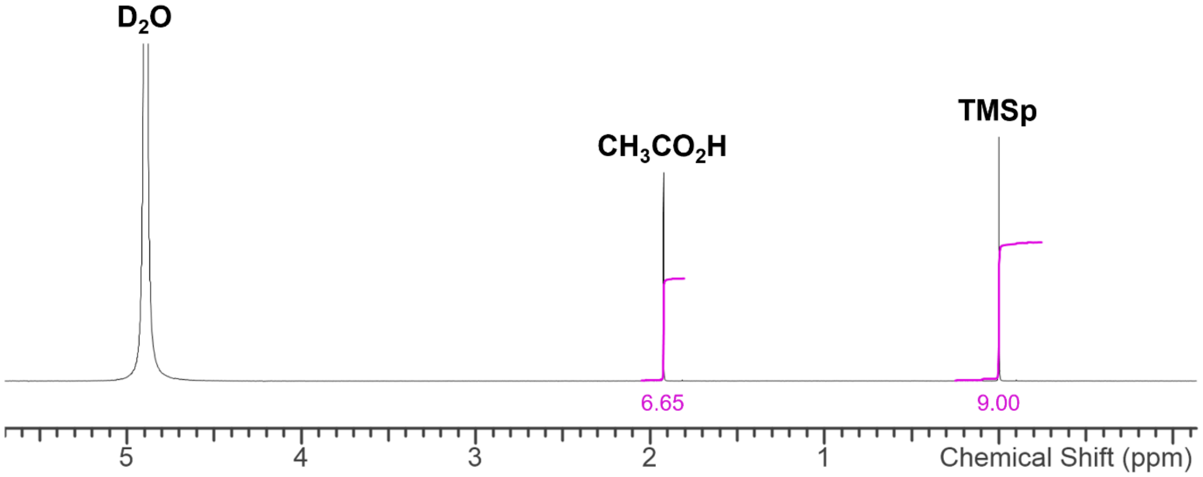

Figure S12 ^{1}H NMR spectrum of 0.8 μmol Hf_{12}(precip) after digestion using 1.0 mL of 1.0 M NH_4HCO_3 with 2.0 mM TMSp-d_4 as internal reference.

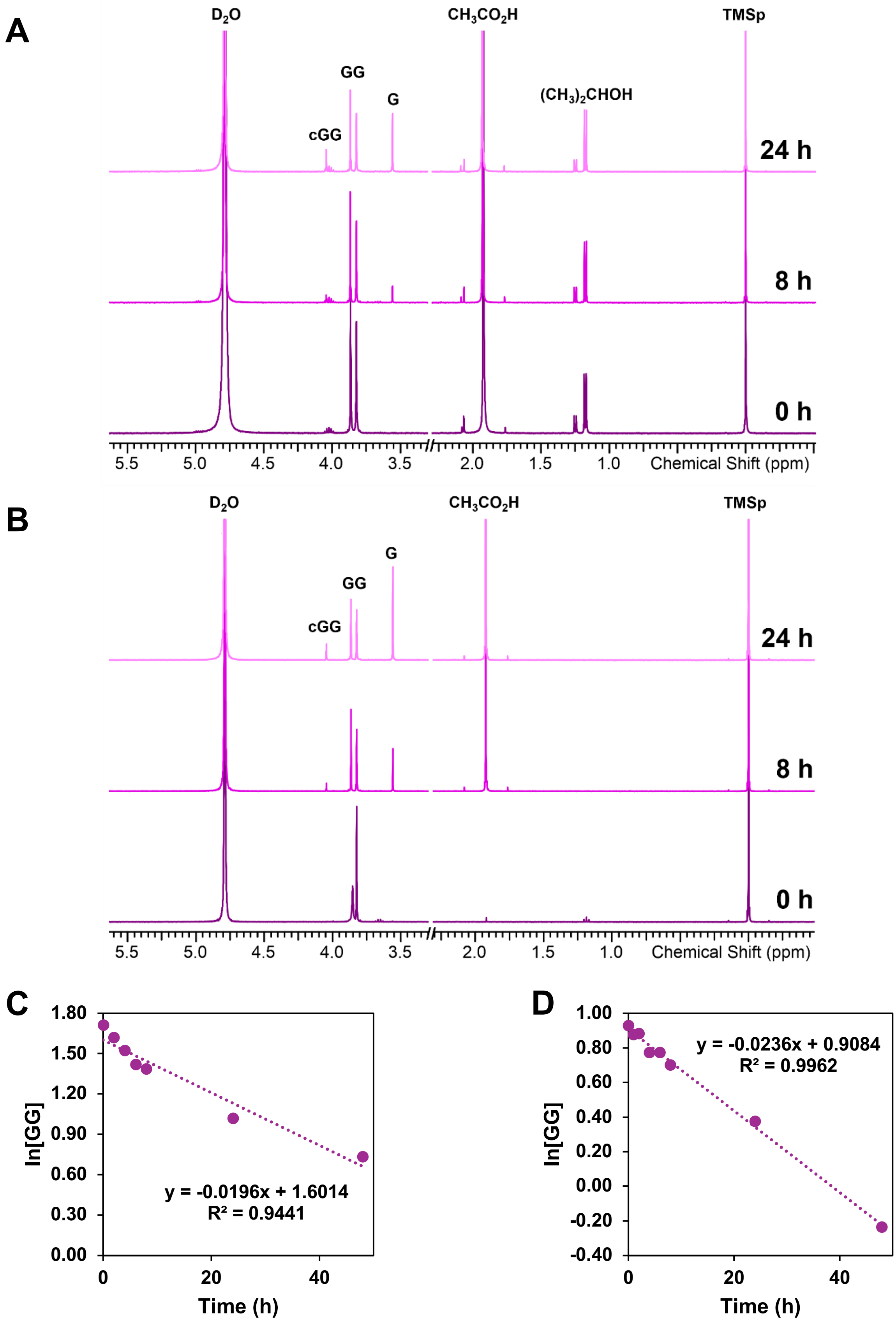

Figure S13 ^{1}H NMR spectra of 2.0 mM GG hydrolysis by an equimolar concentration of (A) Hf_{12}(sol) and (B) Hf_{12}(precip). First-order kinetics of the turnover of GG to G at 60 °C and pD 7.4 by (C) Hf_{12}(sol) and (D) Hf_{12}(precip).

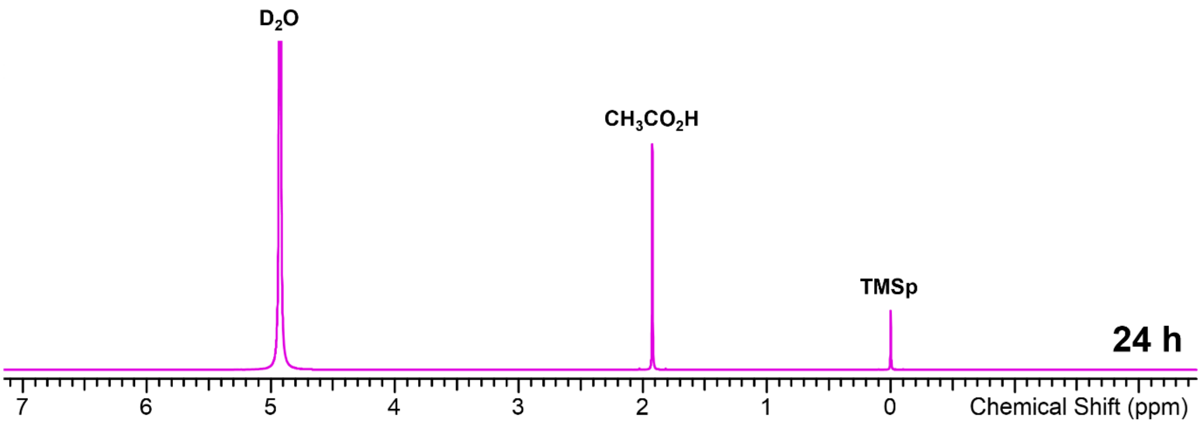

Figure S14 ^{1}H NMR spectrum of supernatant after incubation of 2.0 μmol Hf_{12}(precip) in 1.0 mL D_2O at 60 °C for 24 h.

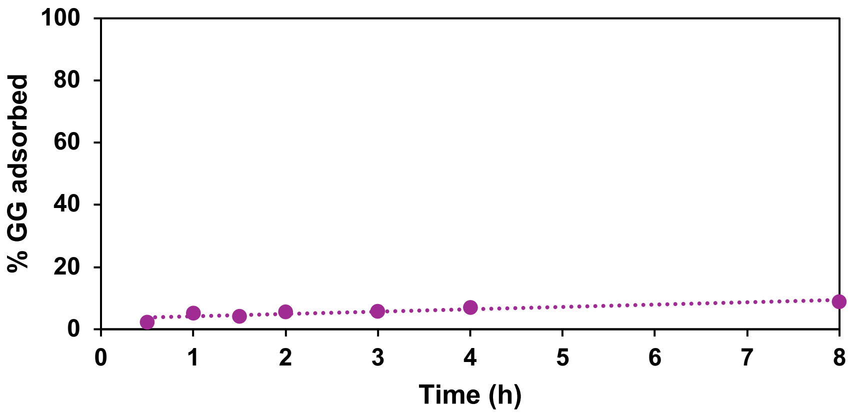

Figure S15 Time-dependent adsorption of 2.0 mM GG on 2.0 μmol Hf_{12}(precip) in 1.0 mL D_2O at 25 °C.

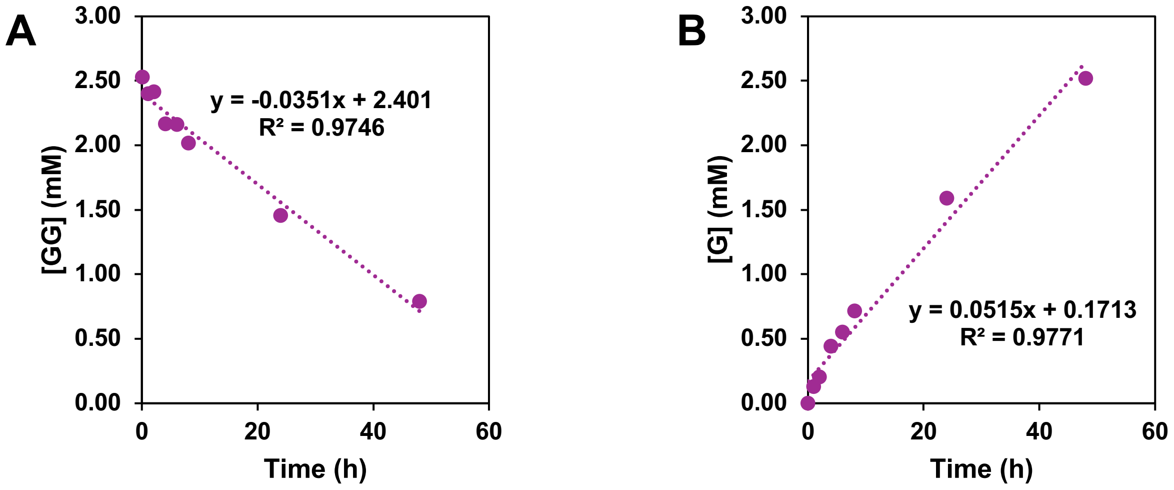

Figure S16 Concentration of (A) GG and (B) G in function of time in presence of Hf_{12}(precip). Based on 0th order kinetics, GG disappears with a rate constant of 9.75×10^{-6} s^{-1} ($t_{½}$ = 19.7 h) whereas G appears with a rate constant of 7.15×10^{-6} s^{-1} ($t_{½}$ = 26.9 h). GG disappears from solution faster than G appears (since two G forms per hydrolyzed GG).

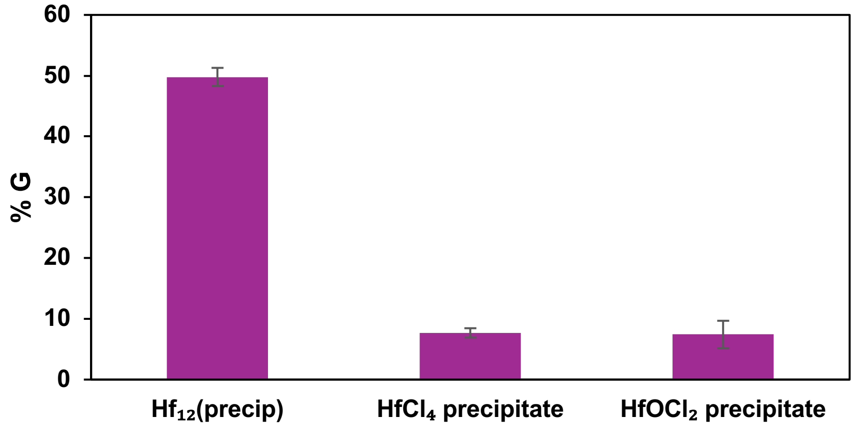

Figure S17 Conversion of 2.0 mM GG to G after 24 h at 60 °C using the precipitates derived from 24.0 mM $HfCl_4$ and $HfOCl_2$, compared to conversion caused by 2.0 μmol Hf_{12}(precip) in 1.0 mL D_2O. Measurements were performed in triplicate. Error bars represent the standard deviation.

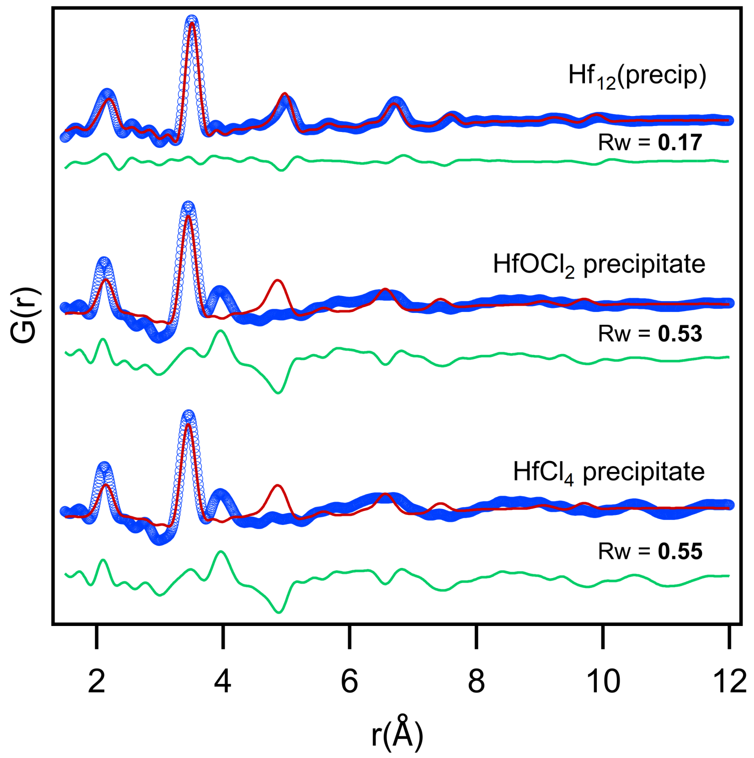

Figure S18 PDF fits of Hf_{12}(precip) and precipitates derived from $HfCl_4$ and $HfOCl_2$ with an oxygen-bridged Hf_{12} model structure extracted from CCDC 2002902.[13]

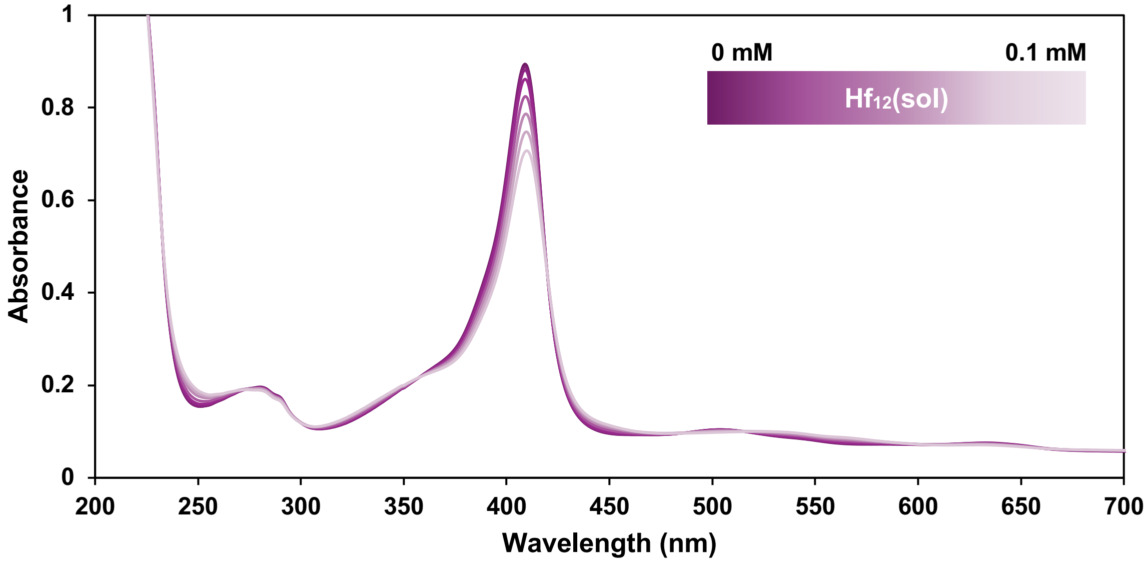

Figure S19 Absorption spectrum of 0.01 mM Mb in the presence and absence of Hf_{12}(sol).

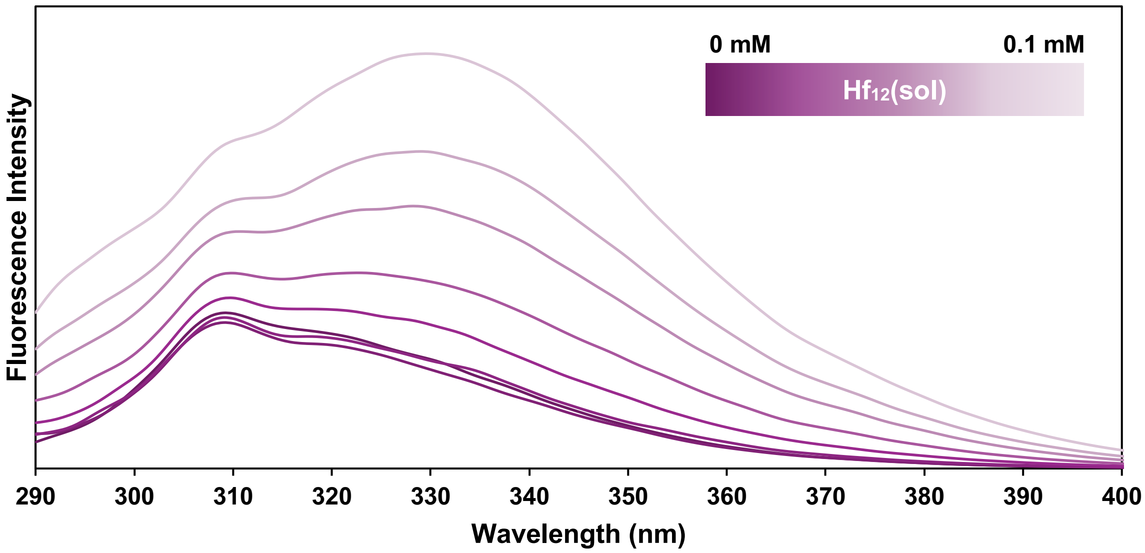

Figure S20 Fluorescence emission spectrum of 0.01 mM Mb in the presence and absence of Hf_{12}(sol).

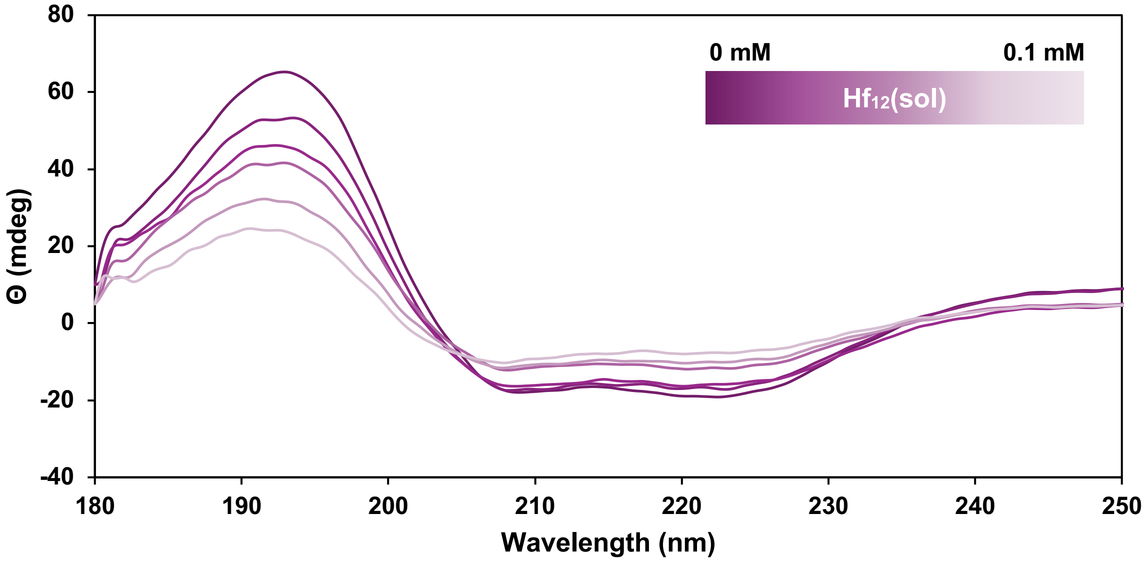

Figure S21 CD spectrum of 0.01 mM Mb in the presence and absence of Hf_{12}(sol).

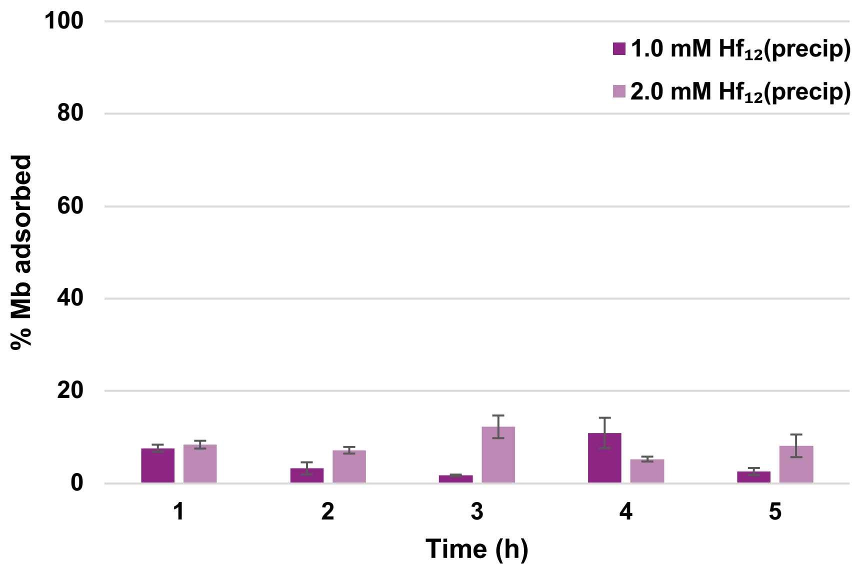

Figure S22 Adsorption of 0.02 mM Mb on 1.0 – 2.0 μmol Hf_{12}(precip) in function of incubation time at 25 °C in 1.0 mL of water. Measurements were performed in triplicate. Error bars represent the standard deviation.

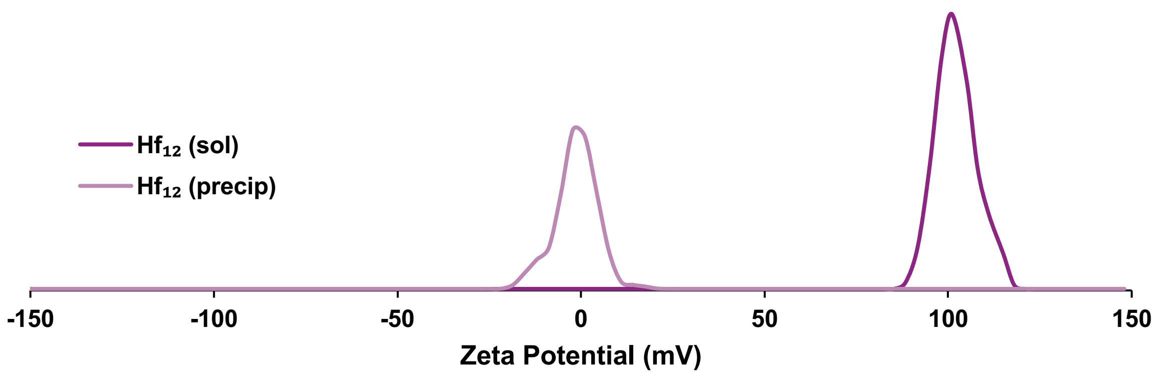

Figure S23 Zeta potential of 2.0 mM Hf_{12}(sol) and 2.0 μmol Hf_{12}(precip) at their native pH in 1.0 mL of water.

Supplementary Tables

Table S1 The Hf(IV) concentration and corresponding % Hf(IV) as fraction of the initial amount of Hf_6 after precipitation of 1.0 mM Hf_{12}(sol) by addition of 1.0 M NaOH to reach pH 8.0 – 12.0.

Precipitation pH	Hf(IV) concentration (ppm)	% Hf(IV) of initial Hf_6
8	0.1830 ± 0.0017	0.0507 ± 0.0005
9	0.0840 ± 0.0006	0.0234 ± 0.0002
10	0.0820 ± 0.0015	0.0229 ± 0.0004
11	0.0800 ± 0.0002	0.0221 ± 0.0001
12	0.0830 ± 0.0014	0.0231 ± 0.0004

Table S2 The Hf(IV) concentration and corresponding % Hf(IV) as fraction of the initial amount of Hf_{12}(precip) after incubation of 1.0 μmol Hf_{12}(precip) in 1.0 mL of water at 60 °C for 144 h at pH 2.0 – 12.0.

Precipitation pH	Hf(IV) concentration (ppm)	% Hf(IV) of initial Hf_{12}(precip)
2	0.0800 ± 0.0021	0.0445 ± 0.0012
3	0.0800 ± 0.0006	0.0445 ± 0.0003
4	0.0760 ± 0.0001	0.0421 ± 0.0001
5	0.0770 ± 0.0015	0.0427 ± 0.0008
7	0.0780 ± 0.0020	0.0433 ± 0.0011
8	0.0870 ± 0.0010	0.0485 ± 0.0006
9	0.0790 ± 0.0013	0.0441 ± 0.0007
10	0.0800 ± 0.0009	0.0443 ± 0.0005
11	0.1370 ± 0.0008	0.0761 ± 0.0004
12	0.0800 ± 0.0022	0.0446 ± 0.0012

Table S3 Refinement parameters for all PDF fits.

Description	Scale	Uiso_Hf	Uiso_C	Uiso_O	delta2	zoom	Rw
**Hf_{12}(sol)**	0.19	0.004	0.017	0.017	3.5	1.000	0.18
**Hf_{12}(precip)**	0.48	0.005	0.090	0.014	3.5	0.997	0.17
**$HfCl_4$ precipitate**	0.054	0.007	0.090	0.010	3.5	0.976	0.55
**$HfOCl_2$ precipitate**	0.058	0.007	0.090	0.011	3.5	0.976	0.53
**Hf_6**	0.042	0.004	0.011	0.038	3.5	0.992	0.26

Table S4 Hydrolysis efficiency across reaction conditions with Hf_{12}(sol) and Hf_{12}(precip) and the relative abundance of individual fragments generated by both clusters (%). Only conditions where all fragments could be properly visualized are given.

Produced Fragments (kDa)	Hf_{12}(sol)				Hf_{12}(precip)	
	5 equiv. 24 h	5 equiv. 144 h	10 equiv. 24 h	10 equiv. 144 h	50 equiv. 24 h	50 equiv. 144 h
16.9	17.6	7.4	12.8	3.6	35.6	32.8
13.4	24.9	17.9	23.0	12.6	32.9	41.5
11.7	1.4	0.8	2.4	1.6	0.9	2.1
10.2	8.9	8.3	4.8	2.1	4.9	3.3
9.0	8.0	7.2	5.9	5.6	–	–
7.6	10.9	7.2	17.8	10.8	16.6	13.6
6.4	–	–	–	–	4.0	1.6
5.1	28.2	51.3	33.3	63.7	5.1	5.0

Table S5 The fragments observed by SDS-PAGE after cleavage of Mb (in kDa) using Hf_{12}(sol) or Hf_{12}(precip) can be attributed to aspartate selective cleavage according to the comparison with fragments obtained for the Zr_6 dimer that were analyzed using LC-MS/MS.

Cleaved Peptide bond	Fragments produced by the Zr_6 dimer (kDa)	Fragments produced by Hf_{12}(sol) (kDa)	Fragments produced by Hf_{12}(precip) (kDa)
D5-G6, F44-D45	4.433	–	–
S109-D110	4.882	5.1	5.1
D5-G6, D61-L62	–	–	6.4
F44-D45, D110-A111	7.414	7.6	7.6
F44-D45, G122-D123	8.705	9.0	–
A20-D21, S109-D110	10.104	10.4	10.2
D5-G6, S109-D110<br>A20-D21, A126-D127	11.714<br>11.785	11.7	11.7
G122-D123	–	13.4	13.4
D21-I22	14.739	–	–

References

[1] S. Dai, C. Simms, I. Dovgaliuk, G. Patriarche, A. Tissot, T. N. Parac-Vogt, C. Serre, *Chem Mater* **2021**, *33* (17), 7057-7066, https://doi.org/10.1021/acs.chemmater.1c02174.

[2] G. Ashiotis, A. Deschildre, Z. Nawaz, J. P. Wright, D. Karkoulis, F. E. Picca, J. Kieffer, *J Appl Crystallogr* **2015**, *48* (2), 510-519, https://doi.org/10.1107/S1600576715004306.

[3] P. Juhás, T. Davis, C. L. Farrow, S. J. L. Billinge, *J Appl Crystallogr* **2013**, *46* (2), 560-566, https://doi.org/10.1107/s0021889813005190.

[4] P. Juhás, C. L. Farrow, X. Yang, K. R. Knox, S. J. Billinge, *Acta Crystallogr A* **2015**, *71* (6), 562-568, https://doi.org/10.1107/S2053273315014473.

[5] R. K. Harris, E. D. Becker, S. M. De Menezes, P. Granger, R. E. Hoffman, K. W. Zilm, P. International Union of, P. Applied Chemistry, D. Biophysical Chemistry, *Magn Reson Chem* **2008**, *46* (6), 582-598, https://doi.org/10.1002/mrc.2225.

[6] R. K. Harris, E. D. Becker, S. M. Cabral de Menezes, R. Goodfellow, P. Granger, *Solid State Nucl Magn Reson* **2002**, *22* (4), 458-483, https://doi.org/10.1006/snmr.2002.0063.

[7] D. Massiot, F. Fayon, M. Capron, I. King, S. Le Calvé, B. Alonso, J. O. Durand, B. Bujoli, Z. H. Gan, G. Hoatson, *Magn Reson Chem* **2002**, *40* (1), 70-76, https://doi.org/10.1002/mrc.984.

[8] J. Pulparayil Mathew, C. Simms, D. E. Salazar Marcano, E. Dhaene, T. N. Parac-Vogt, J. De Roo, *Adv Sci* **2025**, *12* (30), e04713, https://doi.org/10.1002/advs.202504713.

[9] https://www.protpi.ch/Calculator/ProteinTool, accessed: 20-01-2025.

[10] K. Declerck, N. D. Savic, M. A. Moussawi, C. Seno, R. Pokratath, J. De Roo, T. N. Parac-Vogt, *J Am Chem Soc* **2024**, *146* (16), 11400 - 11410, https://doi.org/10.1021/jacs.4c01324.

[11] O. Ernst, T. Zor, *J Vis Exp* **2010**, (38), e1918, https://doi.org/10.3791/1918.

[12] C. Hennig, S. Weiss, W. Kraus, J. Kretzschmar, A. C. Scheinost, *Inorg Chem* **2017**, *56* (5), 2473-2480, https://doi.org/10.1021/acs.inorgchem.6b01624.

[13] J. I. Choi, D. Moon, H. Chun, *Bull Korean Chem Soc* **2020**, *42* (2), 294-302, https://doi.org/10.1002/bkcs.12100.
